# Yeast lunapark regulates the formation of *trans*-Sey1p complexes for homotypic ER membrane fusion

**DOI:** 10.1016/j.isci.2023.108386

**Published:** 2023-11-02

**Authors:** Eunhong Jang, Miriam Lee, So Young Yoon, Sang Soo Lee, Jongseo Park, Mi Sun Jin, Soo Hyun Eom, Changwook Lee, Youngsoo Jun

**Affiliations:** 1 School of Life Sciences, Gwangju Institute of Science and Technology, 123 Cheomdangwagi-ro, Buk-gu, Gwangju 61005, Republic of Korea; 2 Cell Logistics Research Center, Gwangju Institute of Science and Technology, 123 Cheomdangwagi-ro, Buk-gu, Gwangju 61005, Republic of Korea; 3 Department of Biochemistry and Cell Biology, Geisel School of Medicine at Dartmouth, Hanover, NH 03755, USA; 4 Department of Biological Sciences, School of Life Sciences, Ulsan National Institute of Science and Technology, Ulsan 44919, Republic of Korea

**Keywords:** Biochemistry, Molecular biology, Cell biology

## Abstract

The endoplasmic reticulum (ER) consists of the nuclear envelope and a connected peripheral network of tubules and interspersed sheets. The structure of ER tubules is generated and maintained by various proteins, including reticulons, DP1/Yop1p, atlastins, and lunapark. Reticulons and DP1/Yop1p stabilize the high membrane curvature of ER tubules, and atlastins mediate homotypic membrane fusion between ER tubules; however, the exact role of lunapark remains poorly characterized. Here, using isolated yeast ER microsomes and reconstituted proteoliposomes, we directly examined the function of the yeast lunapark Lnp1p for yeast atlastin Sey1p-mediated ER fusion and found that Lnp1p inhibits Sey1p-driven membrane fusion. Furthermore, by using a newly developed assay for monitoring *trans*-Sey1p complex assembly, a prerequisite for ER fusion, we found that assembly of *trans*-Sey1p complexes was increased by the deletion of *LNP1* and decreased by the overexpression of Lnp1p, indicating that Lnp1p inhibits Sey1p-mediated fusion by interfering with assembly of *trans*-Sey1p complexes.

## Highlights

• The yeast lunapark Lnp1p inhibits Sey1p-mediated ER fusion
• *Trans*-Sey1p complexes can be preserved in the presence of GMP-PNP or GDP/AlF_4_^−^
• The N-terminal region of Lnp1p prevents the formation of *trans*-Sey1p complexes

## Graphical abstract

**Figure:**
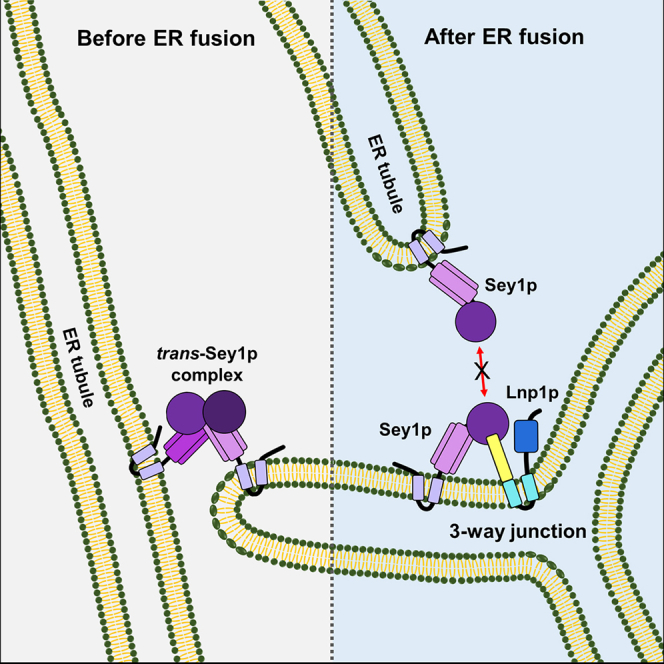

## Introduction

The endoplasmic reticulum (ER) is the largest single organelle in eukaryotic cells and serves as a center for various fundamental cellular functions, including protein translocation and modification, calcium homeostasis, lipid synthesis, and formation of membrane contact sites with other organelles. The ER is a network of sheet-like cisternae and interconnected tubules and is contiguous with the nuclear envelope.^1^^,^^2^^,^^3^ ER sheets assume a flattened sac-like structure, providing a large surface area for ribosome binding. ER tubules spread from peri-nuclear regions to the cell periphery and constantly undergo fusion and fission, which underlies the dynamic structure of the peripheral ER.^4^ They are also involved in regulating the dynamics of other organelles by forming inter-organelle contact sites.^5^^,^^6^ A characteristic of tubular ER is the presence of three-way junctions, which form when the tip of an ER tubule fuses to the side of another ER tubule.^7^ Fusion between ER tubules is mediated by the dynamin-like GTPase atlastin, which is evolutionarily conserved from yeast to human.^8^^,^^9^ A variety of proteins, such as reticulons,^10^^,^^11^ lunapark,^12^ Rab GTPases,^4^^,^^13^ spastin,^14^^,^^15^ protrudin,^16^^,^^17^ and REEP/DP1/Yop1,^11^^,^^18^ are important for maintaining the structure of ER tubules. Among these, lunapark has been suggested to be the only protein that antagonizes the function of atlastins,^12^^,^^19^ although its exact mode of action remains to be elucidated. It has also been reported that lunapark is involved in stabilizing three-way junctions.^20^ Thus, lunapark may fine-tune the complicated structure of the ER by preventing excessive formation of three-way junctions and stabilizing preformed three-way junctions in response to dynamic changes of the intracellular environment. One of the most well-defined functions of lunapark in certain species is as an E3 ligase for atlastins.^21^^,^^22^^,^^23^ Human lunapark possesses ubiquitin ligase activity within its N-terminal cytoplasmic domain and ubiquitinylates atlastin-2.^23^ Atlastin-2 and atlastin-3 are the major atlastin proteins in non-neuronal cells.^24^^,^^25^ The two *Arabidopsis* lunapark proteins, LNP1 and LNP2, physically interact with and ubiquitinylate the *Arabidopsis* atlastin, ROOT HAIR DEFECTIVE3 (RHD3).^22^ Thus, lunapark seems to downregulate atlastin-mediated ER fusion by reducing the cellular levels of atlastin proteins. In the budding yeast *Saccharomyces cerevisiae*, Sey1p, the yeast ortholog of metazoan atlastins, is critical for the formation of three-way junctions of tubular ER, and the yeast lunapark Lnp1p was suggested to function as a negative regulator of Sey1p based on the finding that the collapsed, densely reticulated ER network in *lnp1Δ* is partially restored by the concomitant deletion of Sey1p.^12^ However, the underlying mechanism, including whether Lnp1p functions as a ubiquitin ligase of Sey1p, remains unclear. A clue came from a study by Zhou et al.,^19^ which showed that the N-terminal region of human lunapark inhibits *Drosophila* atlastin-mediated liposome fusion, suggesting that human lunapark directly inhibits atlastin function independently of its ubiquitin E3 ligase activity. Here, we found that Sey1p-mediated ER microsome fusion was markedly increased by the deletion of *LNP1* and decreased by the overexpression of Lnp1p, consistent with the suggested role of lunapark as a negative regulator of atlastin. Furthermore, Lnp1p inhibited Sey1p-mediated liposome fusion when co-reconstituted into liposomes with Sey1p, suggesting that Lnp1p directly prevents Sey1p function. Although the sub-reactions of Sey1p-mediated ER membrane fusion remain poorly characterized, it seems readily accepted that the formation of *trans*-complexes between one Sey1p molecule in one ER tubule and another Sey1p molecule in another ER tubule is a prerequisite for fusion between the two ER tubules. Using a newly developed assay for monitoring the formation of *trans*-Sey1p complexes between two membranes, we demonstrated that Lnp1p reduced the formation of *trans*-Sey1p complexes, which led to the downregulation of Sey1p-mediated fusion.

## Results

### Sey1p-mediated endoplasmic reticulum microsome fusion *in vitro* is increased by the deletion of *LNP1* and decreased by the overexpression of Lnp1p

**Figure 1:**
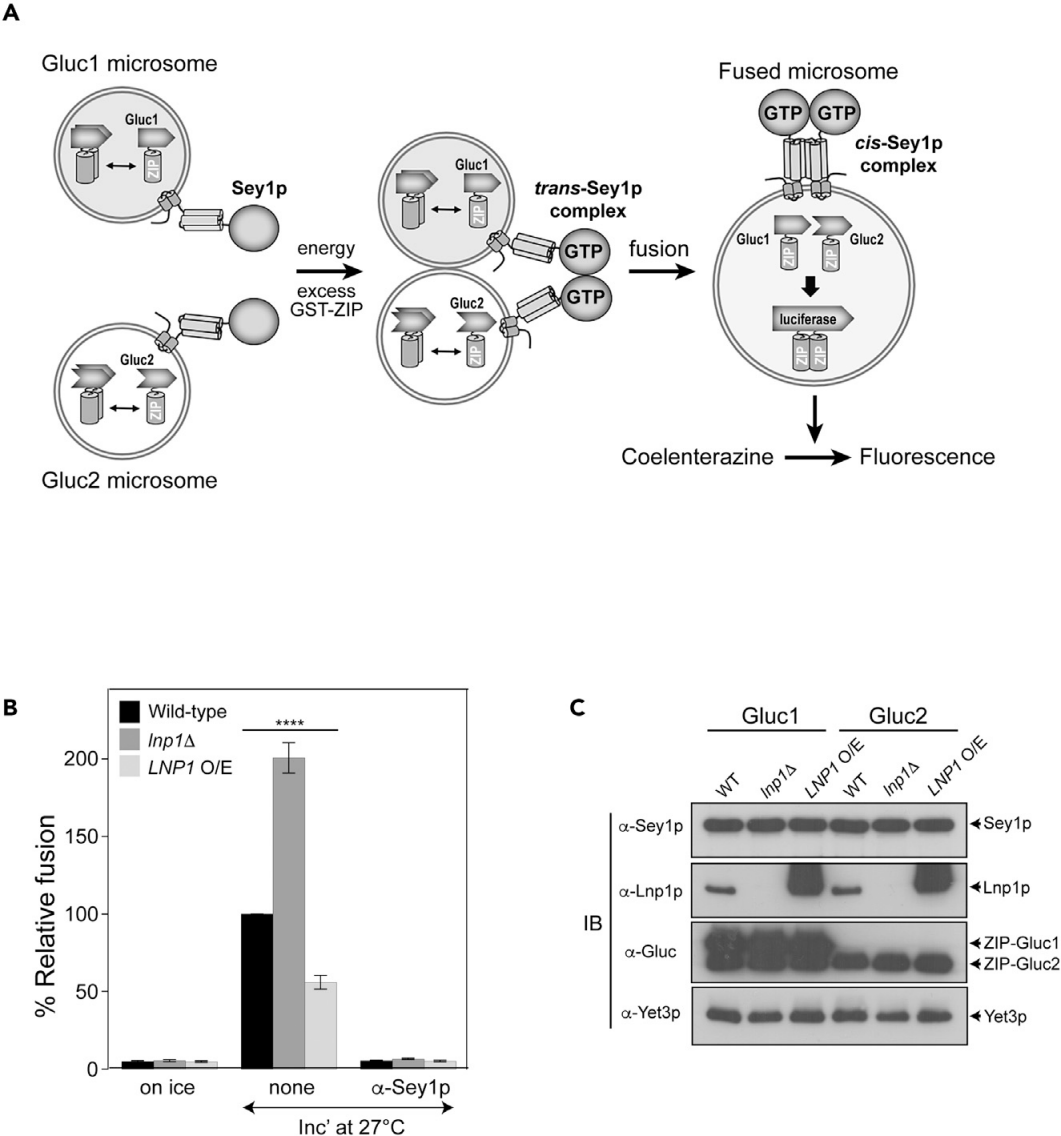
Lnp1p functions as a negative regulator of Sey1p-mediated ER fusion (A) Assay scheme; see results for details. ER microsomes isolated from BJ-Gluc1 yeast cells overexpressing ssZIP-Gluc1-HDEL under the control of the *ADH1* promoter (Gluc1 microsomes) were mixed with microsomes isolated from BJ-Gluc2 cells overexpressing ssZIP-Gluc2-HDEL (Gluc2 microsomes) and then incubated at 27°C in the presence of GTP and ATP. After 90 min, the luciferase substrate coelenterazine was added and luciferase activity was measured. Excess GST-ZIP was added to block the extra-luminal reconstitution of functional Gluc caused by membrane destabilization or rupture during incubation. (B) ER fusion is markedly increased by the deletion of *LNP1* and decreased by its overexpression (O/E). Gluc1 and Gluc2 microsomes were incubated on ice or at 27°C in the absence or presence of α-Sey1p for 90 min. Fusion values were normalized to those obtained using a reaction containing wild-type microsomes alone incubated at 27°C. Data represent the means ± SEM (error bars; n = 3). ∗∗∗∗p < 0.0001, two-way ANOVA with Tukey’s multiple comparisons test. (C) Protein profiles of wild-type, *lnp1*Δ, and *LNP1 O/E* microsomes. Expression of Gluc PCA fragments and ER-associated proteins was analyzed by immunoblotting using the indicated antibodies. The ER-resident protein Yet3p was used as a loading control.

To examine the role of Lnp1p in Sey1p-mediated ER membrane fusion, we employed an *in vitro* fusion assay using ER microsomes isolated from yeast cells.^26^^,^^27^ Briefly, one ER microsome population contains one-half of Gaussia luciferase fused with the zinc-finger (ZIP) domain of GCN4 (Gluc1-ZIP), which readily homodimerizes. The other ER microsome population bears the other half of Gaussia luciferase conjugated with the ZIP domain (Gluc2-ZIP). When these microsomes are mixed and fuse together, content mixing between the two microsome populations allows Gluc1-ZIP and Gluc2-ZIP to interact with each other through ZIP dimerization, reconstituting the activity of Gaussia luciferase. By measuring luciferase activity, membrane fusion between the two microsome populations can be assayed (Figure 1A). To investigate whether Lnp1p is involved in Sey1p-mediated ER fusion, we generated yeast strains in which *LNP1* was deleted or Lnp1p was overexpressed under the control of the *TDH3* promoter, a constitutively strong promoter,^28^ isolated ER microsomes from these strains, and compared their *in vitro* fusion with that between ER microsomes isolated from wild-type yeast cells (Figures 1B and 1C). Fusion was markedly increased by the deletion of *LNP1* and decreased by the overexpression of Lnp1p, indicating that Lnp1p is a negative regulator of Sey1p-mediated ER fusion.

**Figure 2:**
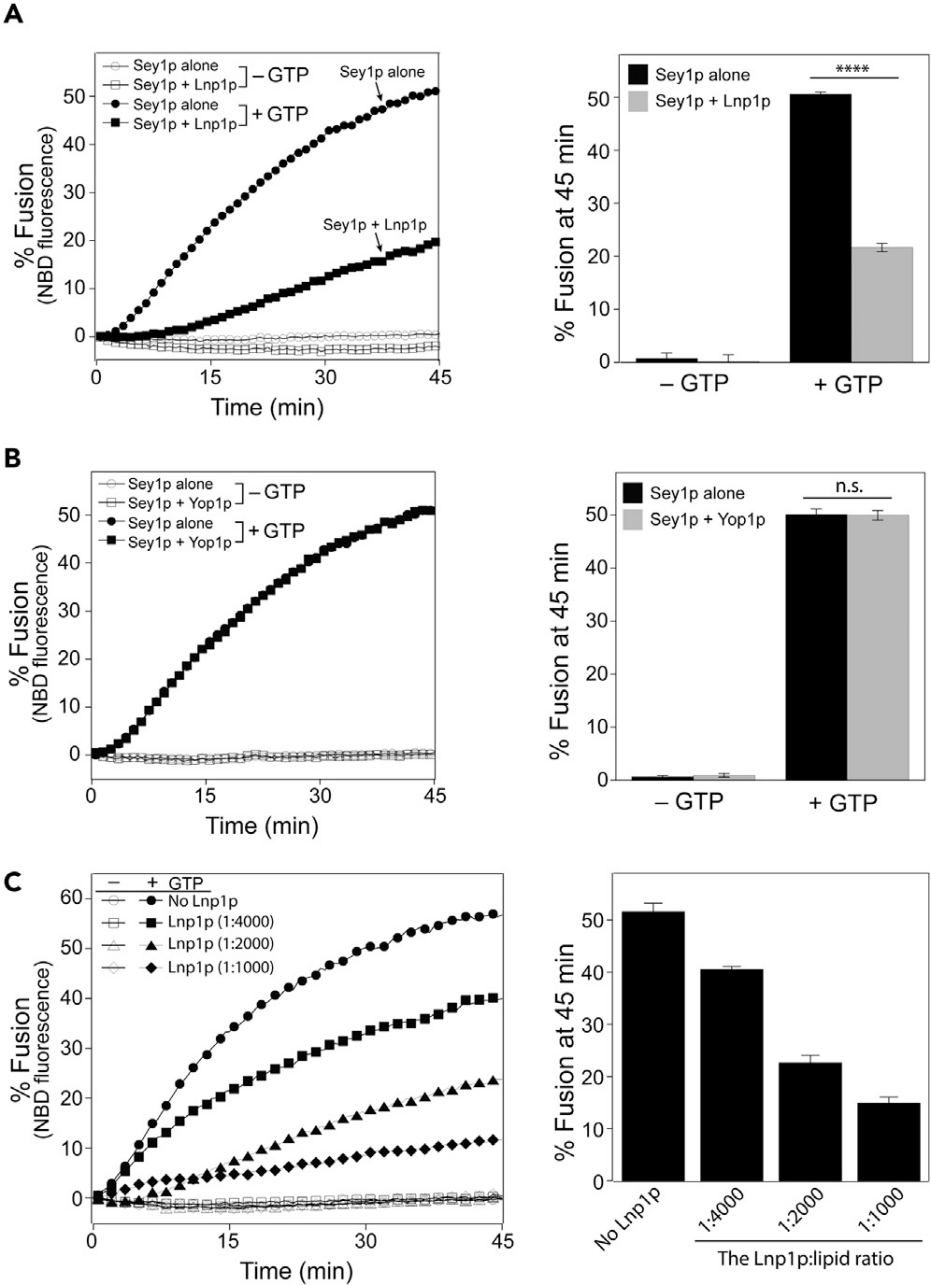
Lnp1p inhibits lipid mixing between Sey1p-containing liposomes (A) Proteoliposomes containing Sey1p alone or both Sey1p and Lnp1p were generated as described in the STAR methods. Donor and acceptor proteoliposomes were mixed and incubated at 30°C for 10 min. After GTP and Mg^2+^ were added, NBD fluorescence was measured at 1 min intervals for 45 min β-octylglucoside was then added to determine total fluorescence. Fusion is expressed as the percentage of total fluorescence. The kinetics graph (left) is representative of three independent results, which are presented in a bar graph (right). Data represent the means ± SEM (error bars; n = 3). ∗∗∗∗p < 0.0001, two-way ANOVA with Tukey’s multiple comparisons test. (B) Yop1p does not affect Sey1p-mediated liposome fusion. (C) Lnp1p inhibits Sey1p-mediated liposome fusion in a dose-dependent manner. Proteoliposomes containing Sey1p alone or both Sey1p and increasing concentrations of Lnp1p were prepared, and donor and acceptor proteoliposomes were mixed and incubated at 30°C for 10 min. After GTP and Mg^2+^ were added, NBD fluorescence was measured at 1 min intervals for 45 min β-octylglucoside was then added to determine total fluorescence. Fusion is expressed as the percentage of total fluorescence. The kinetics graph (left) is representative of three independent results, which are presented in a bar graph (right). Data represent the means ± SEM (error bars; n = 3).

**Figure 3:**
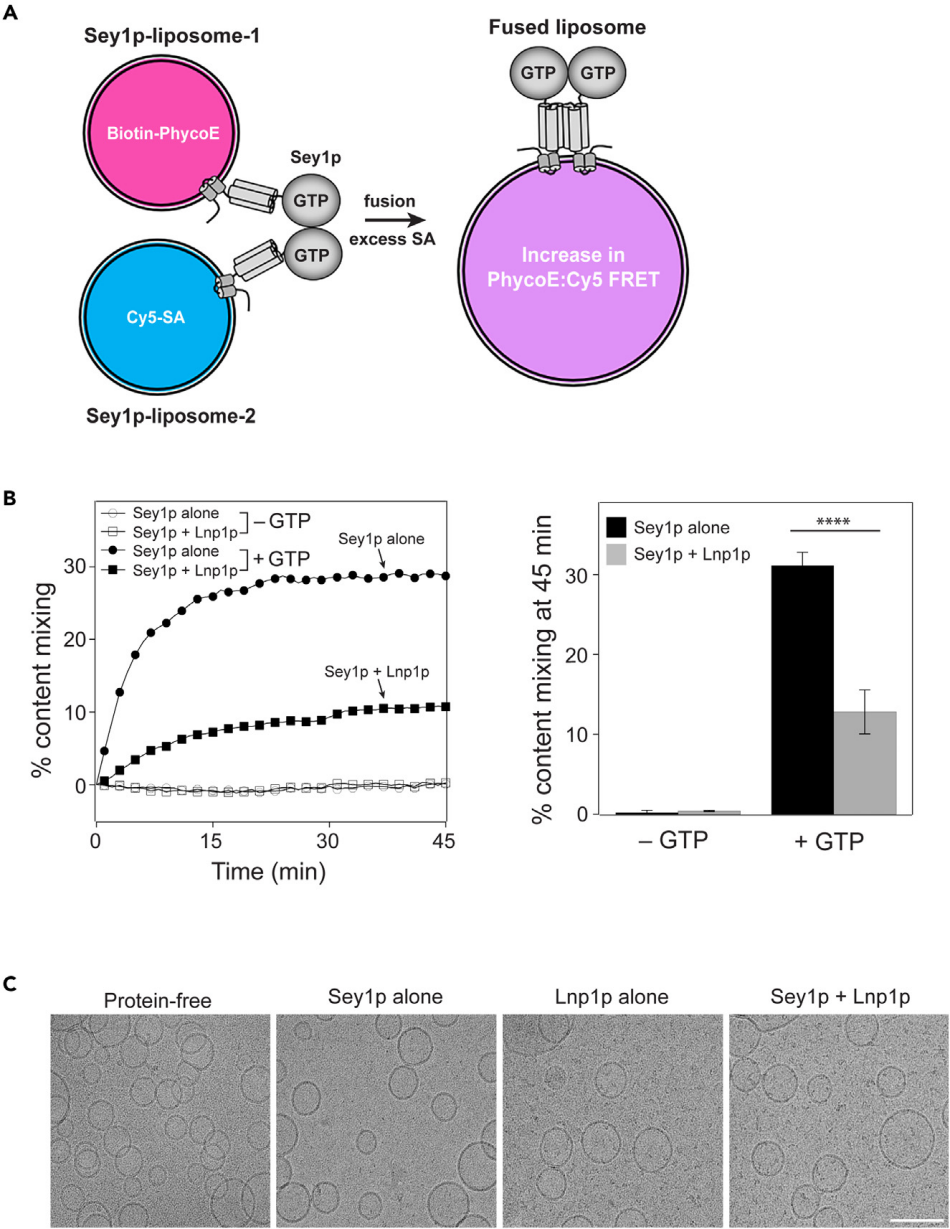
Lnp1p inhibits content mixing between Sey1p-containing liposomes (A) Schematic representation of the content-mixing assay using Sey1p- or Sey1p/Lnp1p-containing liposomes. (B) Sey1p- or Sey1p/Lnp1p-bearing liposomes containing biotin-phycoerythrin (PhycoE) and Sey1p- or Sey1p/Lnp1p-bearing liposomes containing Cy5-SA were mixed and incubated at 30°C for 10 min. Reactions were further incubated in the absence or presence of GTP at 30°C for 45 min, and FRET signals between PhycoE and Cy5 were measured every minute for 45 min. To determine total fluorescence, 1% Thesit was added to the mixture of liposomes containing biotin-PhycoE and liposomes containing Cy5-SA in the absence of streptavidin. The kinetics graph (left) is representative of three independent results, which are presented in a bar graph (right). Data represent the means ± SEM (error bars; n = 3). ∗∗∗∗p < 0.0001, two-way ANOVA with Tukey’s multiple comparisons test. (C) Cryo-EM images of protein-free liposomes, Sey1p-containing liposomes, Lnp1p-containing liposomes, and Sey1p/Lnp1p-containing liposomes. Scale bar: 100 nm.

As shown by the *in vitro* ER microsome fusion assay (Figure 1B), Lnp1p inhibits Sey1p-mediated ER fusion; however, it remains unclear whether the inhibitory activity of Lnp1p requires any additional factors or is achieved by Lnp1p alone. To resolve this issue, we reconstituted Lnp1p into Sey1p-containing liposomes and examined whether the addition of Lnp1p reduced fusion between Sey1p-containing liposomes by measuring lipid mixing between proteoliposomes. We and other research groups previously reported that Sey1p is sufficient to induce liposome fusion.^26^^,^^27^^,^^29^ Fusion between proteoliposomes bearing both Sey1p (1:1000) and Lnp1p (1:2000) was markedly lower than that between liposomes containing Sey1p alone (Figure 2A), confirming that Lnp1p directly inhibits Sey1p-mediated fusion. Addition of Lnp1p during liposome preparation did not affect the amount of Sey1p reconstituted into liposomes (Figure S1). The inhibitory effect of Lnp1p seems to be specific because the reconstitution of Yop1p (1:2000), another ER-shaping protein, into Sey1p-containing liposomes did not affect Sey1p-driven fusion (Figure 2B). This is also consistent with our previous data showing that the deletion of YOP1 little affects ER microsome fusion *in vitro*,^26^ although it was reported that Yop1p at high concentrations (5-fold higher than the concentration of Sey1p) stimulates Sey1p-mediated liposome fusion.^30^ Furthermore, we reconstituted Lnp1p at varying concentrations into Sey1p-containing liposomes (Sey1p:lipid ratio of 1:1000) and found that Lnp1p inhibited Sey1p-mediated fusion in a concentration-dependent manner (Figure 2C). In addition, nearly identical results were obtained in the content-mixing assay, which is based on changes of FRET induced by content mixing (Figure 3A), using proteoliposomes containing both Sey1p and Lnp1p or Sey1p alone (Figure 3B). Thus, our data clearly demonstrate that Lnp1p directly inhibits Sey1p-mediated lipid mixing as well as content mixing. Sey1p belongs to the dynamin-like GTPase family^8^^,^^29^ and its GTPase activity is essential for its fusogenic activity^26^^,^^29^; therefore, Lnp1p may inhibit Sey1p-mediated fusion by preventing the GTPase activity of Sey1p. Lnp1p markedly prevented the GTPase activity of Sey1p when co-reconstituted into liposomes with Sey1p (Figure S2).

A previous study showed that *Xenopus* or human lunapark induces the formation of stacked bilayer discs when reconstituted into liposomes at extremely high concentrations (protein:lipid ratio of 1:200).^31^ If this is also the case for our Lnp1p-containing liposomes, the inhibitory effect of Lnp1p may originate from its ability to convert Sey1p-containing liposomes into stacked discs. To exclude this possibility, we examined the shape of liposomes bearing both Sey1p and Lnp1p, which were used for our *in vitro* fusion reactions, by cryo-electron microscopy (cryo-EM). Liposomes bearing both Sey1p and Lnp1p had a sphere-like shape, similar to protein-free liposomes, liposomes containing Sey1p alone, and liposomes containing Lnp1p alone (Figure 3C). We reconstituted Lnp1p into liposomes at a protein:lipid ratio of 1:2000, whereas 10-fold higher concentrations of *Xenopus* or human lunapark proteins were reported to induce the formation of stacked bilayer discs.^31^ Although lunapark proteins may exist at such a high concentration locally in a certain region of the ER under certain cellular conditions, we estimated that Lnp1p is present in yeast ER microsomes at a protein:lipid ratio of about 1:68,000, which is far lower than the Lnp1p concentration (1:200) required for stacked disc formation.^31^

### Lnp1p inhibits assembly of *trans*-Sey1p complexes

**Figure 4:**
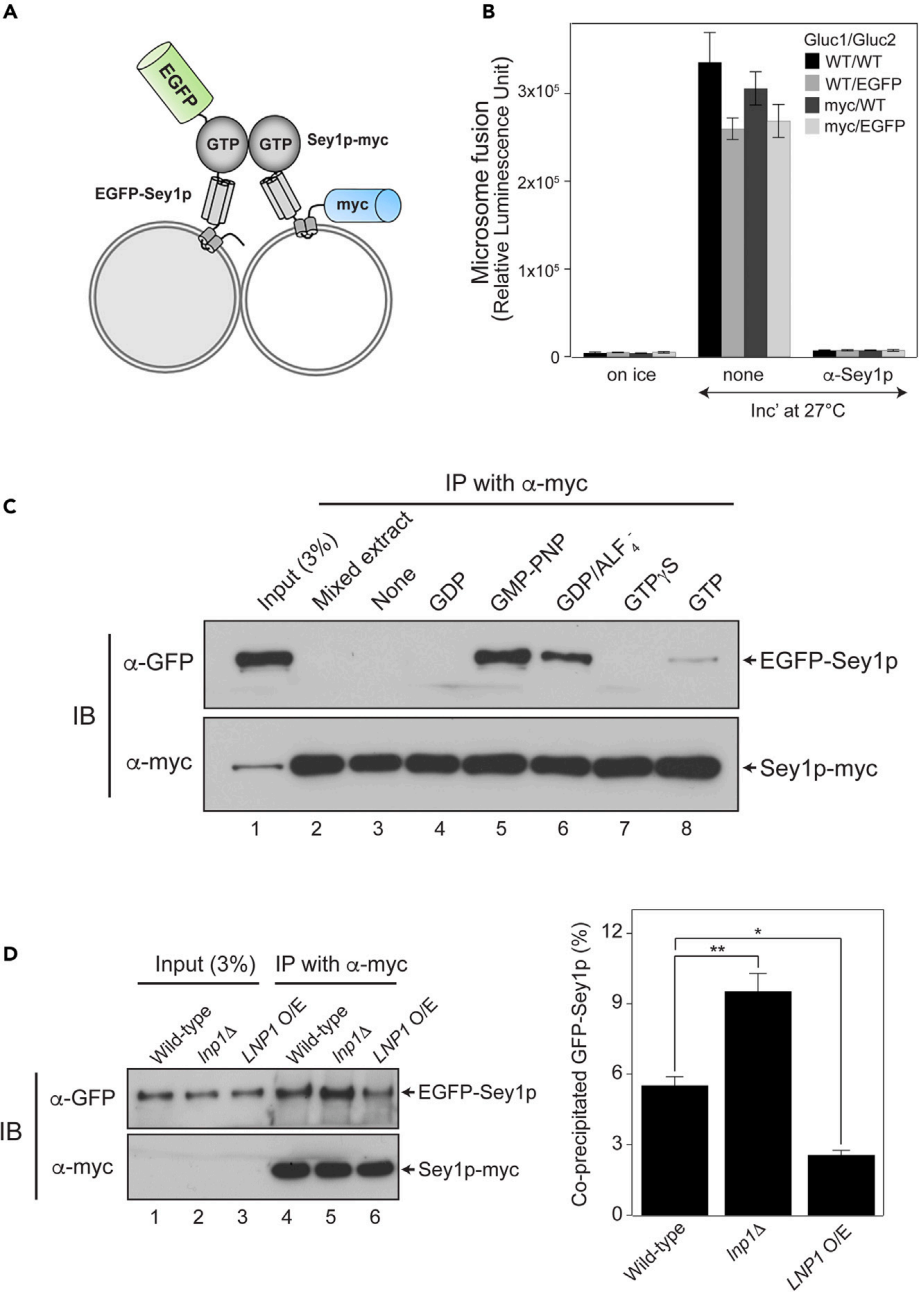
Lnp1p downregulates assembly of *trans*-Sey1p complexes (A) Schematic representation of the *trans*-Sey1p complex formation assay using liposomes bearing EGFP-Sey1p or Sey1p-myc. (B) EGFP-Sey1p and Sey1p-myc support ER microsome fusion comparably with wild-type Sey1p. Data represent the means ± SEM (error bars; n = 3). (C) The *trans*-Sey1p complex formation assay was performed in the absence or presence of GDP, GTP, or GTP analogs. Microsomes isolated from BJ-EGFP-Sey1p were mixed with microsomes purified from BJ-Sey1p-myc and incubated in the absence or presence of GDP, GTP, or GTP analogs at 27°C. After 10 min, membranes were pelleted by centrifugation and solubilized on ice for 20 min. Detergent-insoluble material was removed by centrifugation at 4°C for 10 min, and Sey1p was precipitated using an anti-myc antibody and Protein A Sepharose. Co-precipitated EGFP-Sey1p was analyzed by SDS-PAGE followed by immunoblotting with an anti-GFP antibody. Relative protein levels were estimated by measuring band intensities. (D) Formation of *trans*-Sey1p complexes is markedly increased by the deletion of *LNP1* and decreased by its overexpression (O/E). The immunoblots (left) are representative of three independent results, which are presented in a bar graph (right). Data represent the means ± SEM (error bars; n = 3). ∗p < 0.05, ∗∗p < 0.01, two-way ANOVA with Tukey’s multiple comparisons test.

The data described so far strongly suggest that Lnp1p directly inhibits Sey1p-mediated ER membrane fusion. This led us to examine how Lnp1p prevents Sey1p-mediated fusion. Although the sub-reactions that underlie atlastin-mediated ER membrane fusion are largely uncharacterized, it is generally accepted that assembly of atlastin complexes between two fusing membranes (*in trans*) is a prerequisite for ER fusion.^26^^,^^29^^,^^32^^,^^33^^,^^34^ Thus, we attempted to develop an assay for monitoring the formation of *trans*-Sey1p complexes between two ER membranes (Figure 4A). Briefly, one *sey1*Δ yeast strain was engineered to express myc-tagged Sey1p in the ER and another *sey1*Δ yeast strain was manipulated to have ER containing EGFP-conjugated Sey1p. ER microsomes were isolated from these two strains, mixed, incubated at the physiological temperature in the absence or presence of GTP or its analog, and solubilized with mild detergent. Sey1p-myc was then immunoprecipitated using an anti-myc antibody, and co-precipitated EGFP-Sey1p was analyzed by immunoblotting. Conjugation of myc or EGFP only marginally affected the fusogenic activity of Sey1p (Figure 4B), suggesting that myc-Sey1p and EGFP-Sey1p are largely functional and thus can be used to monitor assembly of *trans*-Sey1p complexes.

Previous studies suggest that the binding of GTP to atlastin is required for the formation of *trans*-atlastin complexes (pre-fusion complexes). After fusion, GTP hydrolysis followed by release of Mg^2+^ and GDP induces disassembly of post-fusion *cis*-atlastin complexes into individual atlastin proteins.^35^^,^^36^ Atlastins present in the same membrane can also undergo GTP-dependent dimerization followed by GTP hydrolysis-induced disassembly into atlastin monomers.^33^ Atlastin dimers can be stabilized in the presence of GDP/AlF_4_, which mimics the transition status of GTP hydrolysis,^32^^,^^34^ and/or GMP-PNP, a non-hydrolyzable GTP analog.^34^ Thus, we tested GDP, GTP, and GTP analogs to determine a condition under which *trans*-Sey1p complexes are best preserved. Incubation of mixtures of detergent-solubilized microsomes expressing Sey1p-myc and EGFP-Sey1p excludes the possibility that *trans*-Sey1p complexes can artifactually form during the incubation of the mixtures of detergent-lysed microsomes, instead of being formed during the fusion reaction (Figure 4C, lane 2). At 10 min after mixing the two populations of microsomes in the absence or presence of GDP, GTP, or its analog at 27°C, we analyzed the formation of *trans*-complexes between Sey1p-myc and EGFP-Sey1p (Figure 4C). As expected, almost no complexes were detected in the absence of guanidine nucleotides (lane 3) or in the presence of GDP (lane 4). In the presence of GTP, only barely detectable amounts of *trans*-Sey1p complexes were observed (lane 8), presumably because these complexes rapidly disassembled into individual Sey1p molecules after GTP hydrolysis.^37^ Consistent with a previous study,^34^ we observed reasonably high amounts of *trans*-Sey1p complexes in the presence of GMP-PNP (lane 5), a non-hydrolyzable GTP, or GDP/AlF_4_ (lane 6), a transition-state analog. Interestingly, GTPγS (lane 7), another non-hydrolyzable GTP analog, failed to preserve any detectable *trans*-Sey1p complexes. Thus, we decided to use GMP-PNP, which preserves the largest amount of *trans*-Sey1p complexes under our experimental condition, to analyze the formation of *trans*-Sey1p complexes throughout this study. The lack of *trans*-Sey1p complex formation during fusion reactions using ER microsomes bearing Sey1p-K50A, a mutant defective in GTP binding or hydrolysis, confirmed that GTP binding is essential for assembly of *trans*-Sey1p complexes (Figure S2). Because ER microsomes expressing Sey1p-K50A do not support fusion,^29^ these data clearly demonstrate that assembly of *trans*-Sey1p complexes correlates well with Sey1p-mediated membrane fusion.

Sey1p-mediated fusion was increased by *LNP1* deletion and decreased by Lnp1p overexpression (Figure 1B); therefore, assembly of *trans*-Sey1p complexes may be similarly affected by the level of *LNP1* expression. The level of *trans*-Sey1p complexes was markedly increased when *LNP1* was deleted (Figure 4D, lane 5) and decreased when Lnp1p was overexpressed (lane 6), again showing a clear positive correlation between the amount of *trans*-Sey1p complexes and the extent of Sey1p-mediated fusion (compare Figures 1B and 4D). These results suggest that Lnp1p functions in Sey1p-mediated ER fusion by regulating assembly of *trans*-Sey1p complexes or earlier steps required for the formation of *trans*-Sey1p complexes during fusion.

**Figure 5:**
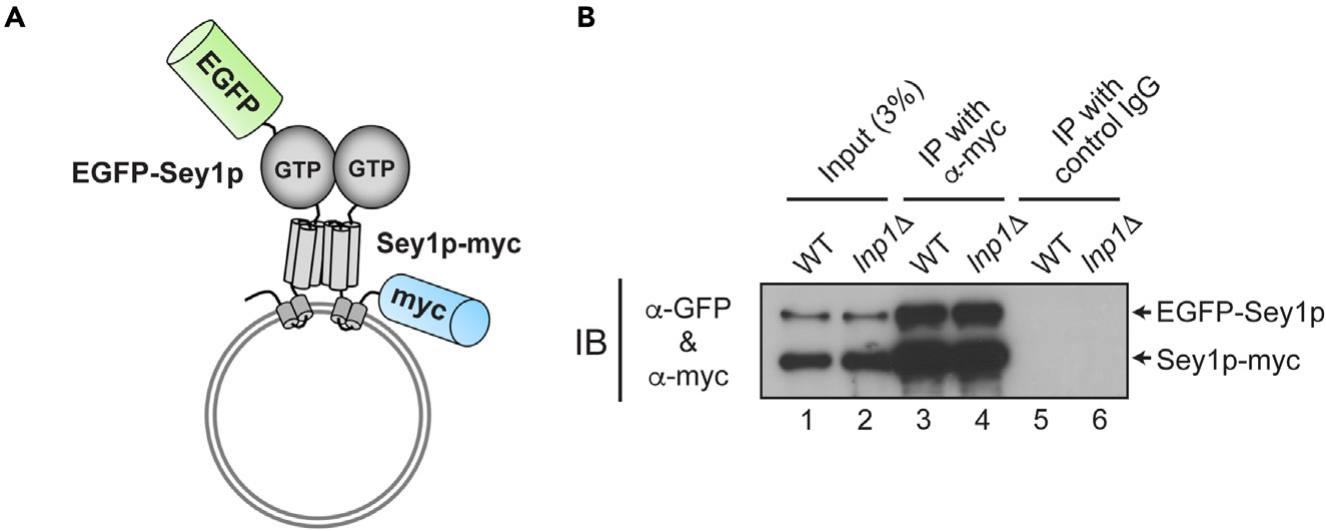
Lnp1p little affects the steady-state level of *cis*-Sey1p complexes (A) Schematic representation of the *cis*-Sey1p complex formation assay using liposomes bearing both EGFP-Sey1p and Sey1p-myc. (B) Deletion of *LNP1* does not influence the steady-state amount of *cis*-Sey1p complexes. Microsomes isolated from BJ-EGFP-Sey1p/Sey1p-myc were solubilized, detergent-insoluble material was removed by centrifugation at 4°C for 10 min, and Sey1p-myc was precipitated using an anti-myc antibody and Protein A Sepharose. Co-precipitated EGFP-Sey1p was analyzed by SDS-PAGE followed by immunoblotting with an anti-Sey1p antibody.

It has been proposed that atlastin proteins in one membrane are not only involved in the formation of *trans*-complexes with atlastin proteins in another membrane, but also associate with each other in the same membrane to form *cis*-complexes.^33^ It was suggested that *cis*-Sey1p complexes need to be dissembled into Sey1p monomers to participate in the formation of *trans*-Sey1p complexes^33^; therefore, the steady-state level of *cis*-Sey1p complexes or individual Sey1p proteins in one membrane is critical for the amount of Sey1p proteins available for assembly of *trans*-Sey1p complexes. Thus, if Lnp1p affects the steady-state level of *cis*-Sey1p complexes, it will also affect the formation of *trans*-Sey1p complexes. To examine whether Lnp1p influences the steady-state level of *cis*-Sey1p complexes, we generated a *sey1*Δ yeast strain that co-expressed Sey1p-myc and EGFP-Sey1p (Figure 5A) and analyzed the amount of *cis*-Sey1p complexes in the presence or absence of Lnp1p by performing co-immunoprecipitation using an anti-myc antibody. Deletion of *LNP1* did not influence the steady-state level of *cis*-Sey1p complexes (Figure 5B), suggesting that the effect of Lnp1p on assembly of *trans*-Sey1p complexes is not via its effect on *cis*-Sey1p complexes.

### The N-terminal region of Lnp1p is responsible for its fusion-inhibitory activity

**Figure 6:**
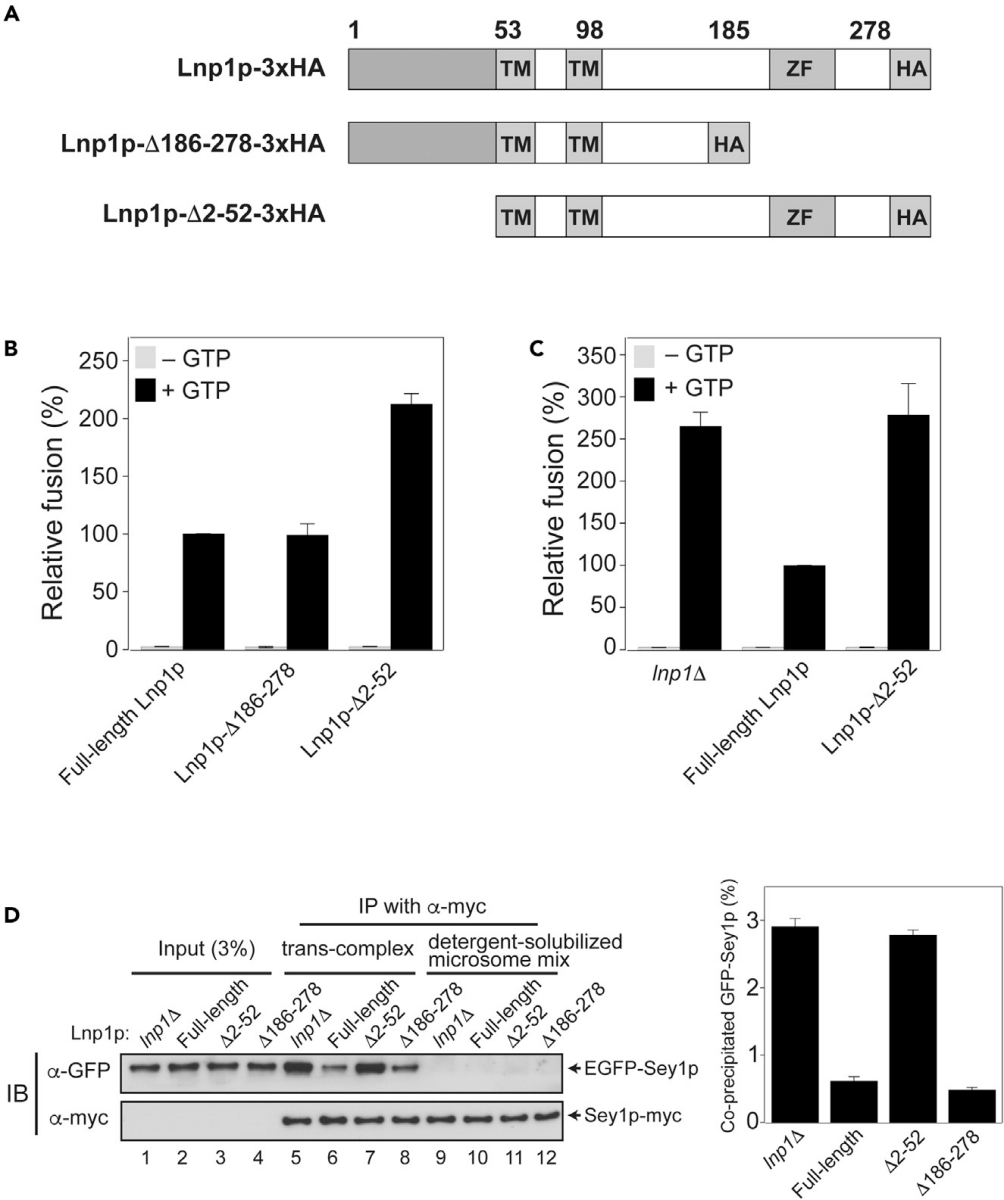
The N-terminal cytoplasmic region of Lnp1p is responsible for its fusion-inhibitory activity (A) Schematic representation of Lnp1p constructs. (B) The N-terminal region of Lnp1p retains its fusion-inhibitory activity. Gluc1 and Gluc2 microsomes isolated from *lnp1*Δ yeast cells bearing 3×HA-tagged full-length Lnp1p, 3×HA-tagged Lnp1p-Δ186–278, or 3×HA-tagged Lnp1p-Δ2–52 were mixed and incubated in the absence or presence of GTP at 27°C for 90 min. (C) Gluc1 and Gluc2 microsomes isolated from *lnp1*Δ yeast cells or *lnp1*Δ yeast cells bearing 3×HA-tagged full-length Lnp1p or 3×HA-tagged Lnp1p-Δ2–52 were mixed and incubated in the absence or presence of GTP at 27°C for 90 min. (D) The amount of *trans*-Sey1p complexes detected from *lnp1*Δ yeast cells is comparable with that detected from *lnp1*Δ yeast cells expressing 3×HA-tagged Lnp1p-Δ2–52. The amount of *trans*-Sey1p complexes was compared among ER microsomes isolated from *lnp1*Δ yeast cells or *lnp1*Δ yeast cells bearing 3×HA-tagged full-length Lnp1p, 3×HA-tagged Lnp1p-Δ186–278, or 3×HA-tagged Lnp1p-Δ2–52.

To elucidate the molecular mechanism by which Lnp1p inhibits the formation of *trans*-Sey1p complexes and thereby Sey1p-mediated fusion, we first attempted to functionally dissect Lnp1p, which contains two transmembrane domains (Figure 6A), allowing both its N- and C-terminal regions to face the cytoplasm. We constructed two Lnp1p mutants, one lacking its zinc-finger motif and the sequence thereafter (Lnp1p-Δ186–278) and another lacking the N-terminal 51 amino acids (Lnp1p-Δ2–52), and compared them with wild-type Lnp1p in the *in vitro* ER microsome fusion assay (Figure 6B and 6C). Lnp1p-Δ186–278 inhibited Sey1p-mediated ER microsome fusion comparably with wild-type Lnp1p; however, ER microsome fusion was markedly increased in the presence of Lnp1p-Δ2–52 (Figure 6B). These results indicate that the N-terminal region retains the fusion-inhibitory activity of Lnp1p. Consistently, the extent of ER microsome fusion in the presence of Lnp1p-Δ2–52 was nearly identical to that observed with *lnp1*Δ microsomes (Figure 6C), further suggesting that the N-terminal region possesses almost all the fusion-inhibitory activity of full-length Lnp1p. To examine whether the fusion-inhibitory activity of the N-terminal region of Lnp1p functions via the inhibition of *trans*-Sey1p complex assembly, we analyzed the formation of *trans*-Sey1p complexes with microsomes expressing wild-type Lnp1p, Lnp1p-Δ186–278, or Lnp1p-Δ2–52. *Trans*-Sey1p complex formation was markedly increased with microsomes bearing Lnp1p-Δ2–52 (lane 7), and the increase was comparable with that observed with *lnp1*Δ microsomes (compare lanes 5 and 7) (Figure 6D). This further supports the idea that the N-terminal region of Lnp1p inhibits Sey1p-mediated ER fusion by preventing assembly of *trans*-Sey1p complexes.

**Figure 7:**
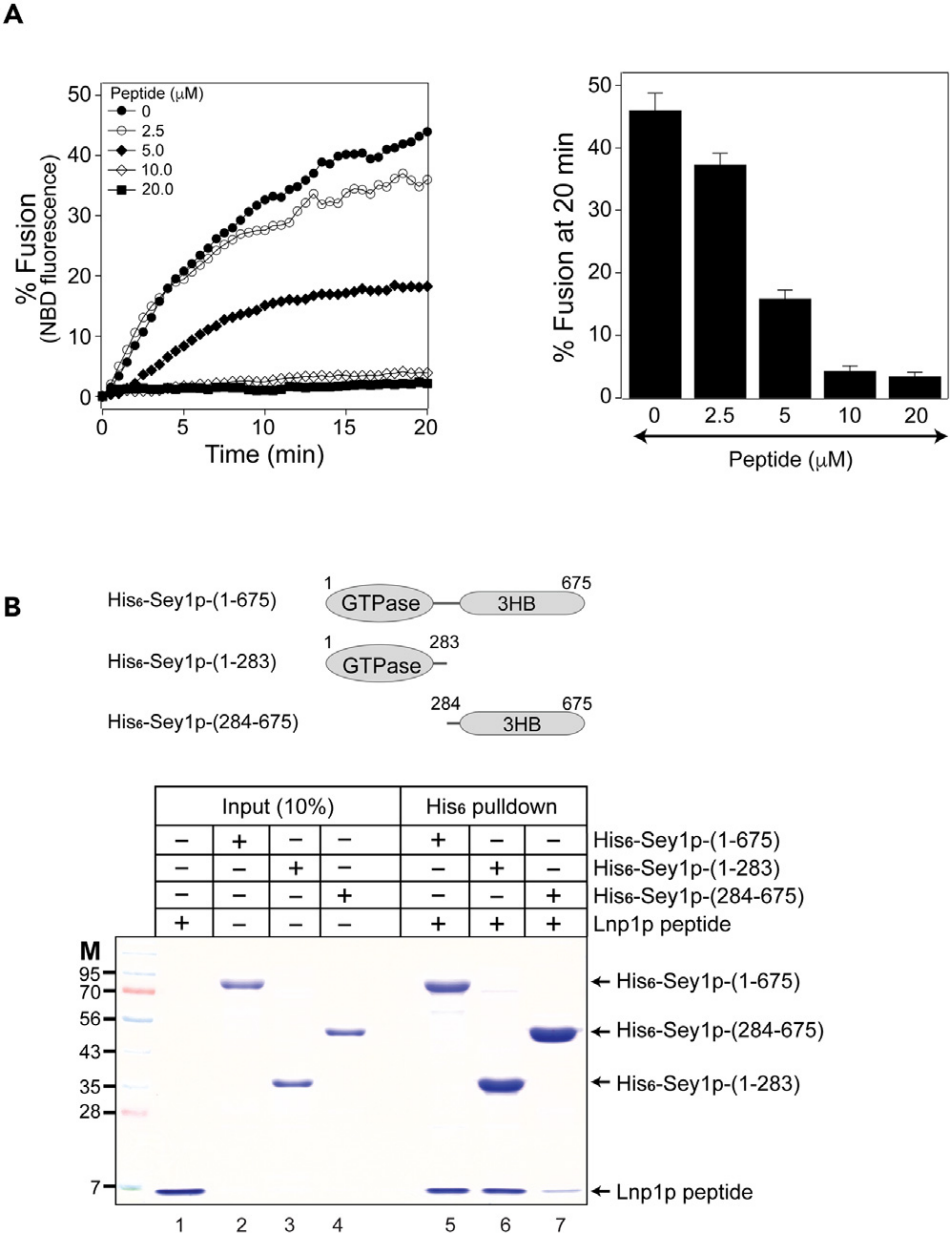
A synthetic peptide derived from the N-terminal region (amino acids 1–52) of Lnp1p inhibits Sey1p-mediated liposome fusion by directly binding to the GTPase domain of Sey1p (A) A peptide containing the N-terminal 52 amino acids of Lnp1p (Lnp1p peptide) inhibits Sey1p-driven liposome fusion in a dose-dependent fashion. Donor and acceptor Sey1p proteoliposomes were mixed and incubated at 30°C for 10 min. After GTP and Mg^2+^ were added, NBD fluorescence was measured at 30 s intervals for 20 min β-Octylglucoside was then added to determine total fluorescence. Fusion is expressed as the percentage of total fluorescence. The kinetics graph (left) is representative of three independent results, which are presented in a bar graph (right). Data represent the means ± SEM (error bars; n = 3). (B) The Lnp1p peptide binds to the GTPase domain of Sey1p. The Lnp1p peptide was incubated with his_6_-tagged Sey1p-(1–675), his_6_-tagged Sey1p-(1–283), or his_6_-tagged Sey1p-(284–675) at 4°C for 2 h. The mixture was then further incubated with Ni-NTA agarose at 4°C for 30 min. Peptides and proteins bound to Ni-NTA were analyzed by SDS-PAGE followed by Coomassie Brilliant Blue staining.

To further confirm that the N-terminal region of Lnp1p directly inhibits Sey1p-mediated fusion, we custom-synthesized a peptide containing the amino acids from the N-terminal region (amino acids 1–52) of Lnp1p and examined whether the addition of this peptide prevented Sey1p-mediated liposome fusion. This peptide inhibited Sey1p-mediated liposome fusion in a dose-dependent manner and nearly complete inhibition was achieved upon the addition of 10 μM of the peptide (Figure 7A), clearly demonstrating that the N-terminal region (amino acids 1–52) of Lnp1p is sufficient to prevent the function of Sey1p. These results are also consistent with a previous study reporting that the N-terminal region of *Drosophila* lunapark prevents atlastin-mediated liposome fusion.^19^ To show that the N-terminal region of Lnp1p inhibits Sey1p function by directly binding to Sey1p, we performed *in vitro* binding experiments (Figure 7B). The N-terminal cytoplasmic region of Sey1p (amino acids 1–675), which includes its GTPase domain and 3 helical bundle (3HB) region, interacted with the Lnp1p peptide (Figure 7B, lane 5). The Lnp1p peptide seemed to preferentially bind to the GTPase domain of Sey1p compared with the 3HB region (compare lanes 6 and 7), consistent with the observation that Lnp1p inhibited the GTPase activity of Sey1p (Figure S2). To examine whether the Lnp1p peptide can directly prevent the dimerization of the GTPase domain of Sey1p, we performed size exclusion chromatography with Sey1p-ΔTM in the presence of the Lnp1p peptide. Sey1p-ΔTM eluted from the column as apparent monomers in the absence of nucleotide (red line), but migrated with a retention time consistent with the homodimer in the presence of GMP-PNP (black line) (Figure S3). As expected, the addition of the Lnp1p peptide markedly decreased the amount of Sey1p-ΔTM dimers in the presence of GMP-PNP (blue line).

## Discussion

Although lunapark has been suggested to be a negative regulator of atlastins, it remains largely unclear how it antagonizes the function of atlastins except that lunapark proteins in some species function as E3 ligases for atlastins. In *Arabidopsis*, double deletion of the two lunapark genes *lnp1* and *lnp2* markedly increases the steady-state level of RHD3 (the plant atlastin) and delays the proteasome-mediated degradation of RHD3, suggesting that plant lunapark proteins antagonize RHD3 by destabilizing it.^22^ Consistently, both *Arabidopsis* LNP1 and LNP2 proteins have ubiquitin ligase activity and LNP1 directly regulates the ubiquitination level of RHD3. Therefore, lunapark proteins promote the degradation of RHD3 through a ubiquitination-based protein degradation pathway that involves these proteins acting as E3 ligases. Mouse LNP1 does not contain any typical E3 ligase domains but possesses E3 ligase activity.^21^ Similarly, human lunapark possesses ubiquitin ligase activity within its N-terminal cytoplasmic domain and ubiquitinylates atlastin-2. Thus, it was reasonable to hypothesize that the yeast lunapark Lnp1p functions as an E3 ligase for the yeast atlastin Sey1p. However, neither deletion nor the overexpression of LNP1 affected the steady-state level of Sey1p compared with wild-type cells (Figure 1C), suggesting that Lnp1p does not antagonize the function of Sey1p by regulating the stability of Sey1p unlike its metazoan and plant cousins. In addition, it has been reported that an N-myristoylation site is largely conserved in the N-termini of lunapark proteins in most metazoan species. This myristoylation seems to be critical for the localization of these proteins to three-way junctions because lunapark protein no longer cluster at three-way junctions when the myristoylation site is mutated.^19^ Interestingly, however, the N-myristoylation site is lacking in Lnp1p, which was reported to localize to three-way junctions,^12^ suggesting that Lnp1p clusters at three-way junctions via a different mechanism than its metazoan orthologs.

Although it is widely accepted that atlastins and their orthologs are major fusogens for membrane fusion between ER tubules, it remains poorly understood how exactly they fuse ER membranes. In particular, assembly of *trans*-atlastin complexes between two fusing ER membranes has never been directly tested using isolated organelles. Several strands of evidence suggest that assembly of *trans*-atlastin complexes is a prerequisite for ER membrane tethering followed by fusion.^32^^,^^33^^,^^34^ Although it remains elusive whether Sey1p behaves similarly to metazoan atlastins, a widely accepted model^35^^,^^36^ proposes that an atlastin molecule from one ER membrane forms a dimer with an atlastin molecule from another ER membrane through their GTPase domains. GTP binding may suffice to trigger a dramatic conformational change in this atlastin dimer (*trans*-complex), which brings the two ER membranes into close proximity, initiating membrane fusion. After fusion, GTP hydrolysis triggers the dissociation of the atlastin dimer into individual atlastin monomers, which become available for the next round of ER membrane fusion. This monomer-dimer cycle is suggested to take a few seconds^37^; therefore, we tested several GTP analogs to determine an experimental condition under which *trans*-Sey1p complexes are best preserved. Previous studies showed that human atlastin-1 can form a dimer in the presence of the non-hydrolyzable GTP analog GMP-PNP or the transition-state analog GDP/AlF_4_^−^.^32^^,^^35^^,^^37^^,^^38^^,^^39^^,^^40^^,^^41^ Similarly, Sey1p (*Candia albican*) dimers form in the presence of GMP-PNP or GDP/AlF_4_^−^.^29^^,^^34^ Consistently, we readily detected *trans*-Sey1p complexes in the presence of GMP-PNP or GDP/AlF_4_^−^, but not GDP. In the presence of GTP, we obtained only barely detectable amounts of *trans*-Sey1p complexes, presumably because these complexes rapidly dissociate upon GTP hydrolysis. Interestingly, in the presence of GTPγS, another non-hydrolyzable GTP analog, no *trans*-Sey1p complexes were observed.

We showed that Lnp1p reduces the amount of *trans*-Sey1p complexes, but does not affect the steady-state level of *cis*-Sey1p complexes. Given that *trans*-Sey1p complexes can become post-fusion *cis*-Sey1p complexes, these data seem to be contradictory. This discrepancy can be explained by the short half-life of *trans*-Sey1p complexes. Only a marginal amount of *trans*-Sey1p complexes was detected in the presence of GTP, which was markedly lower than that detected in the presence of GMP-PNP (Figure 4C). Thus, upon GTP hydrolysis, *trans*-Sey1p complexes seem to rapidly dissociate into individual Sey1p molecules. Interestingly, however, about 30% of Sey1p proteins seem to exist in *cis*-complexes at the steady-state. These data collectively suggest that most *cis*-Sey1p complexes are not post-fusion *cis*-Sey1p complexes, which are derived from *trans*-Sey1p complexes, but likely form by the association of Sey1p monomers in the same membrane. Previously, it was reported that atlastins can oligomerize in the same membrane via their transmembrane segments in a nucleotide-independent manner.^42^ Consistently, the formation of *cis*-Sey1p complexes does not require GTP binding. Sey1p-K50A, a mutant defective for GTP binding that did not support assembly of *trans*-Sey1p complexes (Figure S4), fully supported assembly of *cis*-Sey1p complexes (Figure S5). Our data suggest that the N-terminal region of Lnp1p inhibits assembly of *trans*-Sey1p complexes through its direct association with the GTPase domain of Sey1p; therefore, Lnp1p is unlikely to affect transmembrane segment-mediated assembly of *cis*-Sey1p complexes. This idea is consistent with the observation that the deletion of *LNP1* did not affect the steady-state level of *cis*-Sey1p complexes (Figure 5).

Based on the data presented in this study, together with those reported in previous studies^12^^,^^19^^,^^20^^,^^33^ regarding the interplay between atlastin and lunapark during ER membrane fusion, we propose a working model of how Lnp1p stabilizes preformed three-way junctions by preventing excessive Sey1p-mediated fusion (Figure S6). Sey1p monomers from one ER tubule interact with Sey1p monomers from another ER tubule to form *trans*-Sey1p complexes in a GTP-dependent manner. GTP binding triggers drastic conformational changes in each Sey1p molecule in *trans*-Sey1p complexes, which eventually results in membrane fusion between the two ER tubules, generating a three-way junction. At this newly formed three-way junction, upon GTP hydrolysis, post-fusion *cis*-Sey1p complexes are rapidly disassembled into Sey1p monomers, which re-associate to form *cis*-Sey1p complexes via their transmembrane interactions in a GTP-independent manner. Alternatively, Sey1p monomers interact with Lnp1p through the association between the GTPase domain of Sey1p and the N-terminal region of Lnp1p at or near the three-way junction, which prevents Sey1p monomers from forming *trans*-Sey1p complexes with Sey1p monomers from another ER tubule. In this way, Lnp1p prevents excessive Sey1p-mediated fusion near newly formed three-way junctions, eventually stabilizing preformed three-way junctions.

### Limitations of the study

A potential limitation of this study relates to the use of myc- and EGFP-tagged Sey1p for monitoring the assembly of *trans*-Sey1p and *cis*-Sey1p complexes. Although we showed that myc- and EGFP-tagged Sey1p supported ER microsome fusion *in vitro* comparably with their untagged versions, we cannot completely rule out the possibility that the epitope tagging affects the dynamics of Sey1p complexes during ER membrane fusion. Furthermore, this study did not employ a Sey1p mutant equivalent to *Drosophila* atlastin (D127N), a GTP hydrolysis mutant; therefore, we could not distinguish whether GTP binding or hydrolysis is sufficient to trigger the formation of *trans*-Sey1p complexes.

## STAR★Methods

### Key resources table

**Table:** 

REAGENT or RESOURCE	SOURCE	IDENTIFIER
**Antibodies**		

Mouse monoclonal anti-myc	Cell Signaling Technology	Cat#2276S; RRID:AB_331783
Rabbit polyclonal anti-GFP	Thermo Fisher Scientific	Cat#A-11122; RRID:AB_221569
Rabbit monoclonal anti-HA	Cell Signaling Technology	Cat#3724S; RRID:AB_1549585
Rabbit polyclonal anti-Gluc	New England Biolabs	Cat#E8023S; RRID:AB_1929564
Rabbit polyclonal anti-Sey1p	Abfrontier (custom antibody)	
Rabbit polyclonal anti-Lnp1p	Abfrontier (custom antibody)	
Rabbit polyclonal anti-Yet3p	Provided by Charles Barlowe (Geisel School of Medicine at Dartmouth) (Lee et al.)^26^	

**Bacterial and virus strains**		

Rosetta(DE3) competent cells	Novagen	Cat#70954

**Chemicals, peptides, and recombinant proteins**		

Isopropyl β-D-1-thiogalactopyranoside (IPTG)	Gold Biotechnology Inc,	Cat#I2481C
Coelenterazine	Gold Biotechnology Inc,	Cat#55779-48-1
GTP (Guanosine 5′-triphosphate, Lithium salt)	Roche	Cat#11140957001
GDP (Guanosine 5′ diphosphate, Sodium salt)	Sigma-Aldrich	Cat#43139-22-6
GMP-PNP(GppNHp)	Jena Bioscience	Cat#NU-401-50
GTPγS (Guanosine-5'-(γ-thio)-triphosphate, Tetralithium salt)	Jena Bioscience	Cat#NU-412-10
Pyruvate Kinase (PK)	Roche	Cat#10128155001
L-Lactate Dehydrogenase (L-LDH)	Roche	Cat#10127876001
Phosphoenolpyruvate (PEP)	Roche	Cat#10152960103
NADH	Roche	Cat#10107735001
18:2 phosphatidylcholine (PC)	Avanti Polar Lipids	Cat#850385C-25mg
18:2 phosphatidylethanolamine (PE)	Avanti Polar Lipids	Cat#850755C-25mg
18:2 phosphatidylserine (PS)	Avanti Polar Lipids	Cat#840040P-25mg
18:2 phosphatidic acid (PA)	Avanti Polar Lipids	Cat#840885C-25mg
18:1 NBD PE	Avanti Polar Lipids	Cat#810145P-5mg
18:1 Liss Rhod PE	Avanti Polar Lipids	Cat#810150C-5mg
Ergosterol	Sigma-Aldrich	Cat#45480
Cy5-conjugated streptavidin	Vector Laboratories Inc,	Cat#SA-1500-1
R-Phycoerythrin, Biotin-XX Conjugate	Thermo Fisher Scientific	Cat#P811
LNP1 peptide	Anygen (custom peptide)	
His_6_−tagged Sey1p	(Lee et al.)^26^	
His_6_−tagged TEV protease	(Lee et al.)^26^	
His_6_−tagged Sey1p−(1-675)	This paper	
His_6_−tagged Sey1p−(1-283)	This paper	
His_6_−tagged Sey1p−(284-675)	This paper	
His_6_−tagged Yop1p	This paper	
GST-tagged Lnp1p	This paper	

**Deposited data**		

Raw Data of Figures	Mendeley	https://doi.org/10.17632/3c8g6vknrg.1

**Experimental models: Organisms/strains**		

Please refer to Table S1		

**Recombinant DNA**		

Plasmid: pET28a-Sey1p	(Lee et al.)^26^	pET28a Novagen Cat#69864
Plasmid: pGST-ZIP	(Lee et al.)^26^	pGST-Parallel1 (Sheffield et al.)^43^
Plasmid: pYJ406-ssZIP-GLuc1-HDEL	(Lee et al.)^26^	
Plasmid: pYJ406-ssZIP-GLuc2-HDEL	(Lee et al.)^26^	
Plasmid: pET28a-Sey1p−(1-675)	This paper	pET28a Novagen Cat#69864
Plasmid: pET28a-Sey1p−(1-283)	This paper	pET28a Novagen Cat#69864
Plasmid: pET28a-Sey1p−(284-675)	This paper	pET28a Novagen Cat#69864
Plasmid: pGST-Parallel1-Lnp1p	This paper	pGST-Parallel1 (Sheffield et al.)^43^
Plasmid: pHIS-Parallel1-Yop1p	This paper	pHIS-Parallel1 (Sheffield et al.)^43^
Plasmid: pYJ404-Sey1p-myc	This paper	
Plasmid: pYJ406-Sey1p-myc	This paper	pYJ406 (Starai et al.)^44^
Plasmid: pYJ406-EGFP-Sey1p	This paper	pYJ406 (Starai et al.)^44^
Plasmid: pYJ408-EGFP-Sey1p	This paper	pYJ408 (Lee et al.)^26^
Plasmid: pYJ406-Sey1p-K50A-myc	This paper	pYJ406 (Starai et al.)^44^
Plasmid: pYJ408-EGFP-Sey1p-K50A	This paper	pYJ408 (Lee et al.)^26^
Plasmid: pRS408-pTDH3-Lnp1p	This paper	
Plasmid: pYJ408-Lnp1p-3xHA	This paper	pYJ408 (Lee et al.)^26^
Plasmid: pYJ408-Lnp1p-□186-278-3xHA	This paper	pYJ408 (Lee et al.)^26^
Plasmid: pYJ408-Lnp1p-□2-52-3xHA	This paper	pYJ408 (Lee et al.)^26^

**Software and algorithms**		

MikroWin 2000	Berthold Technologies	https://mikrowin-2000.software.informer.com/
Gemini XPS Soft Max pro	Molecular Devices	https://www.moleculardevices.com
Quantity One (basic)	Bio-Rad	https://www.bio-rad.com/ko-kr/product/quantity-one-1-*d*-analysis-software
GraphPad Prism 9	GraphPad	https://www.graphpad.com/

**Other**		

Centro LB 960 Microplate Luminometer	Berthold Technologies	https://www.berthold.com/
Gemini XPS	Molecular Devices	https://www.moleculardevices.com/
BioSpectrometer	Eppendorf	https://www.eppendorf.com/
Beckman Optima L-90K Ultracentrifuge	BECKMAN COULTER	https://www.beckmancoulter.com/

### Resource availability

#### Lead contact

• Further information and requests for resources should be directed to and will be fulfilled by the lead contact: Youngsoo Jun (junys@gist.ac.kr).

#### Material availability

• This study did not generate new unique reagents. Strains and plasmids generated in this study are available on request to the lead contact.

#### Data and code availability

• All data reported in this paper will be shared by the lead contact upon request.
• This paper does not report original code.
• Any additional information required to reassess the data reported in this study is available from the lead contact upon request.

### Experimental model and study participant details

#### Yeast strains

The yeast strains used in this study are listed in Table S1. BJ-Gluc1, BJ-Gluc2, and their derivatives^26^ were used for the *in vitro* ER fusion assay. To assay formation of *trans*-Sey1p complexes, BJ3505 *sey1Δ* was transformed with BsaI-linearized pYJ406-Sey1p-myc or pYJ406-EGFP-Sey1p, generating BJ-Sey1p-myc or BJ-EGFP-Sey1p, respectively. To assay formation of *cis*-Sey1p complexes, BJ3505 *sey1Δ* was transformed with both BsaI-linearized pYJ406-Sey1p-myc and pYJ408-EGFP-Sey1p, generating BJ-Sey1-myc/EGFP-Sey1p. To generate *lnp1Δ* strains, the *LNP1* gene was deleted by PCR-mediated gene disruption.^45^ To generate strains overexpressing Lnp1p, yeast cells were transformed with SnaBI-linearized pRS408-pTDH3-Lnp1p.

### Method details

#### DNA constructs and plasmids

DNA fragments encoding Lnp1p and Sey1p were PCR-amplified from purified yeast genomic DNA. The plasmids pYJ404-Sey1p-myc and pYJ406-Sey1p-myc were generated by inserting DNA fragments encoding Sey1p-myc into pYJ404 and pYJ406, respectively. The plasmids pYJ406-EGFP-Sey1p and pYJ408-EGFP-Sey1p were generated by inserting DNA fragments encoding EGFP, which was PCR-amplified from pEGFP-N3 (Clontech), and Sey1p into pYJ406^44^ and pYJ408,^26^ respectively. The plasmids pYJ406-Sey1p-K50A-myc and pYJ408-EGFP-Sey1p-K50A were generated by site-directed mutagenesis of pYJ406-Sey1p-myc and pYJ408-EGFP-Sey1p, respectively, and the mutations were confirmed by sequencing. To overexpress Lnp1p, pRS408-pTDH3-Lnp1p was generated by replacing the DNA fragment encoding Sey1p in pRS408-pTDH-Sey1p^26^ with a DNA fragment encoding Lnp1p. DNA fragments encoding full-length Lnp1p, Lnp1p-Δ186–278, and Lnp1p-Δ2–52 were PCR-amplified, conjugated with a DNA fragment encoding a 3xHA tag, and inserted into pYJ408 to generate pYJ408-Lnp1p-3xHA, pYJ408-Lnp1p-Δ186-278-3xHA, and pYJ408-Lnp1p-Δ2-52-3xHA, respectively. DNA fragments encoding Sey1p, Sey1p-(1–675), Sey1p-(1–283), and Sey1p-(284–675) were PCR-amplified and inserted into pET28a (Novagen) to generate pET28a-Sey1p, pET28a-Sey1p-(1–675), pET28a-Sey1p-(1–283), and pET28a-Sey1p-(284–675), respectively. DNA fragments encoding Yop1p and Lnp1p were PCR-amplified and inserted into pHIS-Parallel1^43^ and pGST-Parallel1^43^ to generate pHIS-Yop1p and pGST-Lnp1p, respectively.

#### Antibodies

Anti-Sey1p rabbit polyclonal antibodies were previously described.^26^ Anti-Lnp1p rabbit polyclonal antibodies were custom-raised against the C-terminal cytosolic domain (amino acids 100–278) of Lnp1p, which was conjugated with GST (AbFrontier). These antibodies were affinity-purified using their corresponding antigen-bound SulfoLink resins (Thermo Fisher Scientific) and dialyzed against PS buffer (10 mM Pipes-KOH, pH 6.8, and 200 mM sorbitol) containing 125 mM KCl. Anti-myc mouse monoclonal (Cell Signaling Technology), anti-GFP mouse monoclonal (Invitrogen), anti-HA rabbit monoclonal (Cell Signaling Technology), and anti-Gluc rabbit polyclonal (New England Biolabs) antibodies were purchased. Anti-Yet3p rabbit sera were provided by C. Barlowe (Geisel School of Medicine at Dartmouth).

#### Preparation of ER microsomes

ER microsomes were prepared as described previously,^26^ with modifications. In brief, yeast cells were grown in YPD to an OD600 of 1.5–2.0 and collected by centrifugation at 4400*g* for 3 min at 25°C. Cells were then resuspended in 100 mM Tris-HCl, pH 9.4, containing 10 mM DTT and incubated at 30°C for 10 min. Cells were collected and resuspended in spheroplasting buffer (0.1% yeast extract, 0.2% bacteriological peptone, 0.2% glucose, 50 mM KPO_4_, and 600 mM sorbitol, pH 7.5) containing lyticase (0.45 mg/mL). After incubation at 30°C for 35 min, spheroplasts were collected by centrifugation at 1700 *g* at 4°C for 3 min and gently resuspended in ice-cold 15% Ficoll solution (15% Ficoll, 10 mM Pipes-KOH, pH 6.8, and 200 mM sorbitol), and 300 μL of 10 mg/mL DEAE-dextran solution prepared in PS-buffered 15% Ficoll was added. The suspension was incubated on ice for 3 min and then at 30°C for 3 min. Microsomes were isolated by floatation through a discontinuous Ficoll step gradient, which was accomplished by transferring the suspension (5 mL) to an SW40 tube (Beckman Coulter), overlaying it with 2 mL of buffered 8% Ficoll, 4 mL of buffered 4% Ficoll, and 2 mL of PS buffer, and centrifuging it at 180,000 g for 90 min at 4°C. Microsomes were collected from the 4%/8% interface.

#### *In vitro* ER microsome fusion assay

The standard ER microsome fusion reaction (50 μL) contained 5 μg of Gluc1 microsomes, 5 μg of Gluc2 microsomes, reaction buffer (10 mM Pipes-KOH, pH 6.8, 125 mM KCl, 5 mM MgCl_2_, and 200 mM sorbitol), an energy-regenerating system (1 mM MgCl_2_, 1 mg/mL creatine kinase, and 29 mM creatine phosphate), 10 μM coenzyme A, 1 mM ATP, 1 mM GTP, and 100 μM GST-ZIP. Fusion mixtures were incubated at 27°C. After 90 min, 30 μL of the reaction mixture was mixed with 30 μL of coelenterazine (40 μM, GoldBio) and transferred to a 96-well white plate for measurement of luminescence using a luminometer (Centro XS^3^ LB960, Berthold Technologies).

#### *Trans*-Sey1p complex formation assay

The standard *trans*-Sey1p complex formation assay reaction (400 μL) contained 50 μg of microsomes isolated from BJ-Sey1p-myc, 50 μg of microsomes purified from BJ-EGFP-Sey1p, and reaction buffer (10 mM Pipes-KOH, pH 6.8, 125 mM KCl, 5 mM MgCl_2_, and 200 mM sorbitol) containing 1 mM GDP, GTP, GTPγS, GMP-PNP, or GDP/AlF_4_^−^. After incubation at 27°C for 10–45 min, membranes were pelleted by centrifugation at 11,000 g at 4°C for 10 min. After the supernatant was removed, the pellet was washed with ice-cold reaction buffer, resuspended in ice-cold solubilization buffer (25 mM HEPES-NaOH, pH 7.4, 150 mM NaCl, 0.4% Triton X-100, 1 mM PMSF, and 10 μM leupeptin), and incubated on ice for 20 min. Detergent-insoluble material was removed by centrifugation at 11,000 g at 4°C for 10 min. The resulting supernatant was mixed with an anti-myc antibody and incubated on a rotator at 4°C for 6 h. After Protein A Sepharose (Invitrogen) was added, the mixture was further incubated at 4°C for 1 h. Protein A Sepharose beads were collected by centrifugation at 3000 *g* at 4°C for 1 min and washed with ice-cold solubilization buffer three times. Proteins bound to beads were eluted by boiling in SDS sample buffer and analyzed by immunoblotting using anti-myc and anti-GFP antibodies. Relative protein levels were estimated by measuring band intensities (Quantity One, Bio-Rad).

#### Preparation of recombinant proteins

All recombinant proteins used in this study were purified from *Escherichia coli* Rosetta-2 DE3 (Novagen). Expression of recombinant proteins was induced by growing transformed bacteria in LB medium containing 0.5 mM IPTG at 16°C for 14 h. Cells were harvested by centrifugation, resuspended in ice-cold Buffer A (25 mM HEPES-NaOH, pH 7.4, 200 mM NaCl, 10% glycerol, 1 mM EDTA, 1 mM DTT, and 1 mM PMSF) containing a protease inhibitor cocktail (Roche), and lysed by sonication. After centrifugation at 200,000 g at 4°C for 30 min, the pellet was homogenized in Buffer B (25 mM HEPES-NaOH, pH 7.4, 200 mM NaCl, 10% glycerol, 0.5 mM EDTA, and 1% Triton X-100) and incubated with gentle agitation at 4°C for 30 min. The homogenate was then centrifuged at 200,000 g at 4°C for 30 min, and the resulting supernatant was mixed with Ni-NTA beads (QIAGEN) for preparation of his_6_-tagged proteins or with glutathione beads (GE Healthcare) for preparation of GST-tagged proteins at 4°C for 2 h. Protein bound to the beads were washed with Buffer C (20 mM HEPES-NaOH, pH 7.4, 150 mM NaCl, 10% glycerol, 0.5 mM EDTA, and 0.4% Triton X-100) three times and eluted with Buffer C containing 400 mM imidazole or 20 mM reduced glutathione for his_6_-or GST-tagged proteins, respectively. Eluates were concentrated using a centrifugal filter tube (Sigma-Aldrich) and dialyzed against RB150 buffer (20 mM HEPES-NaOH, pH 7.4, 150 mM NaCl, and 10% glycerol) containing 1 mM EDTA and 0.4% Triton X-100.

#### Proteoliposome reconstitution and lipid-mixing assay

All non-fluorescent lipids were purchased from Avanti Polar Lipids except for ergosterol (Sigma-Aldrich). The fluorescent lipids NBD-PE and N-(lissamine rhodamine B sulfonyl)-PE (Rh-PE) were purchased from Invitrogen. ER-mimicking lipid mixes^46^ for proteoliposomes contained 18:2 phosphatidylcholine (47% mol/mol), 18:2 phosphatidylethanolamine (17% or 19% for donor or acceptor liposomes, respectively), 18:2 phosphatidylserine (18%), 18:2 phosphatidic acid (3%), ergosterol (10%), diacylglycerol (1%), cardiolipin (1%), and fluorescent lipids (1.5% NBD-PE and 1.5% Rh-PE for donor liposomes; 1% dansyl-PE for acceptor liposomes). These lipids in chloroform were dried under a stream of nitrogen gas and then further dried using SpeedVac (Thermo Fisher Scientific). The dried lipid mixes were dissolved in 2.5× concentrated RB150 buffer containing 125 mM n-octyl-β-D-glucopyranoside (β-OG) by nutation at room temperature for 2 h. The resulting lipid solution was mixed with protein at the indicated molar ratio of protein:lipid and incubated at 4°C for 2 h. The mixture was dialyzed against RB150 buffer containing 1 mM EDTA in the presence of Bio-Beads SM-2 (Bio-Rad) at 4°C for 16 h, generating proteoliposomes. Proteoliposomes were then incubated with Bio-Beads SM-2 and tobacco etch virus (TEV) protease at 4°C for 2 h. After further incubation in the presence of Bio-Beads SM-2 at 4°C for 4 h, proteoliposomes were mixed with 70% [w/v] Histodenz (Sigma-Aldrich), transferred to an ultracentrifuge tube, overlaid with 25% Histodenz, and topped with RB150 buffer containing 1 mM EDTA. The sample was then centrifuged at 250,000 g at 4°C for 1 h, and proteoliposomes were collected, adjusted to a final lipid concentration of 2 mM with RB150 buffer containing 1 mM EDTA, aliquoted, frozen in liquid nitrogen, and stored at −80°C. The lipid-mixing assay was performed as previously described^26^^,^^46^ with minor modifications. Donor and acceptor liposomes were mixed at a molar ratio of 1:5 in a black 384-well plate with 1 mM MgCl_2_ and incubated at 30°C for 10 min. The reaction was initiated by adding 1 mM GTP and 1 mM MgCl_2_, and the NBD signal was measured every 30 s (excitation at 460 nm, emission at 538 nm) for 30 min using a SpectraMAX Gemini XPS plate reader (Molecular Devices). After reactions, β-OG was added to determine the total NBD fluorescence, and fusion (%) was expressed as the percentage of total NBD fluorescence.

#### Content-mixing assay

ER-mimicking lipid mixes were prepared and dissolved in 2.5× concentrated RB150 buffer containing 125 mM β-OG as described above. The resulting lipid solution was then mixed with the indicated proteins, TEV protease, and biotinylated phycoerythrin (4 μM) for donor liposomes or Cy5-conjugated streptavidin (8 μM). The mixture was dialyzed against RB150 buffer containing 1 mM EDTA with Bio-Beads SM-2 at 4°C for 16 h. Proteoliposomes were obtained by floatation via density gradient centrifugation using Histodenz as described above. Proteasomes were collected, adjusted to a final lipid concentration of 2 mM with RB150 buffer containing 1 mM EDTA, aliquoted, frozen in liquid nitrogen, and stored at −80°C. For the content-mixing assay, donor (0.25 mM) and acceptor (0.25 mM) proteoliposomes were mixed with 1 mM MgCl_2_ and 5 μM streptavidin in a black 384-well plate and incubated at 30°C for 10 min. Reactions were initiated by adding 1 mM GTP and 1 mM MgCl_2_, and the FRET signal between phycoerythrin and Cy5 (excitation at 565 nm, emission at 670 nm) was measured every minute for 30 min using a SpectraMAX Gemini XPS plate reader. Total fluorescence was estimated by adding 1% [w/v] Thesit to the proteoliposome mixture in the absence of streptavidin.

#### Cryo-EM sample preparation and imaging

Cryo-EM samples were produced using Vitrobot Mark IV (FEI) at 4°C and 100% humidity. Briefly, 3 μL of 2 mM liposomes were loaded into glow-discharged 300-mesh Cu R 1.2/1.3 Holey carbon grids (Quantifoil). After the grids were blotted using Whatman filter paper for 2 s in a humidified atmosphere and plunge-frozen in liquid ethane, cryo-EM images were collected using a 200 kV Glacios transmission electron microscope (FEI) with a Falcon 3 direct electron detector (FEI). Images were manually captured at a nominal magnification of 92,000 (corresponding to a pixel size of 0.83 Å). The total exposure dose was 30e^−^/Å^2^.

#### Protein-peptide pull-down experiments

In total, 40 μg of his_6_-tagged proteins was mixed with 20 μM Lnp1p peptide in RB150 buffer (500 μL) containing 0.4% Triton X-100 and 20 mM imidazole at 4°C for 2 h. The protein-peptide mixtures were then further incubated with Ni-NTA agarose in RB150 buffer containing 0.4% Triton X-100 and 20 mM imidazole at 4°C for 30 min. After three washes with RB150 buffer containing 0.4% Triton X-100 and 20 mM imidazole, proteins bound to Ni-NTA agarose were eluted by boiling in SDS sample buffer and analyzed by SDS-PAGE and Coomassie Brilliant Blue staining.

#### GTPase activity measurement

The GTPase activity of Sey1p was measured using a continuous, regenerative coupled GTPase assay.^47^ In brief, 1 mM Sey1p-containing proteasomes (1 μM Sey1p) were added to 100 μL of reaction buffer (20 mM HEPES-NaOH, pH 7.4, and 150 mM NaCl) containing 6 μg of pyruvate kinase (Roche), 3.2 μg of lactate dehydrogenase (Roche), 4 mM phosphophenolpyruvate (Roche), 0.3 mM NADH, 2 mM GTP, and 10 mM MgCl_2_. Depletion of NADH through its oxidation, which is directly proportional to GTP hydrolysis, was measured by monitoring a decrease in absorbance at 340 nm at 30°C every 30 s for 10 min using a spectrometer (BioSpectrometer, Eppendorf) through a 1 cm light path.

#### Size exclusion chromatography

To assess the interaction between Sey1p-ΔTM and the Lnp1p peptide, purified proteins were incubated in the absence or presence of the Lnp1p peptide and GMP-PNP at 30°C for 1 h, and the mixture was injected into a Superdex 200 (10/300) column (GE Healthcare) equilibrated with 20 mM HEPES-NaOH, pH 7.4, and 150 mM NaCl. Sey1p-ΔTM proteins were monitored by absorbance at 280 nm, and the entire elution peaks were analyzed by SDS-PAGE followed by Coomassie Brilliant Blue staining.

### Quantification and statistical analysis

Statistical significance was determined from at least three independent experiments using GraphPad Prism 9 (GraphPad Software) by a one- or two-way ANOVA with Tukey’s test for multiple comparisons. The data distribution was assumed to be normal, but this was not formally tested. All data represent the mean ± SEM. For all statistical tests, p values are denoted as follows: ∗∗∗∗p < 0.0001; ∗∗∗p < 0.001; ∗∗p < 0.01; ∗p < 0.05. Details on the experimental n and on the number of replicates are included for each experiment in the corresponding figure legend.
