# Supplementary material for: Yeast lunapark regulates the formation of trans-Sey1p complexes for homotypic ER membrane fusion
Source: iScience. 2023 Nov 2;26(12):108386. doi: 10.1016/j.isci.2023.108386 (PMC10679814; doi:10.1016/j.isci.2023.108386)
Supplement: Document S1. Figures S1–S6 and Table S1 [file mmc1.pdf]

iScience, Volume *26*

Supplemental information

Yeast lunapark regulates the formation of *trans*-Sey1p complexes for homotypic ER membrane fusion

Eunhong Jang, Miriam Lee, So Young Yoon, Sang Soo Lee, Jongseo Park, Mi Sun Jin, Soo Hyun Eom, Changwook Lee, and Youngsoo Jun

Supplemental Information

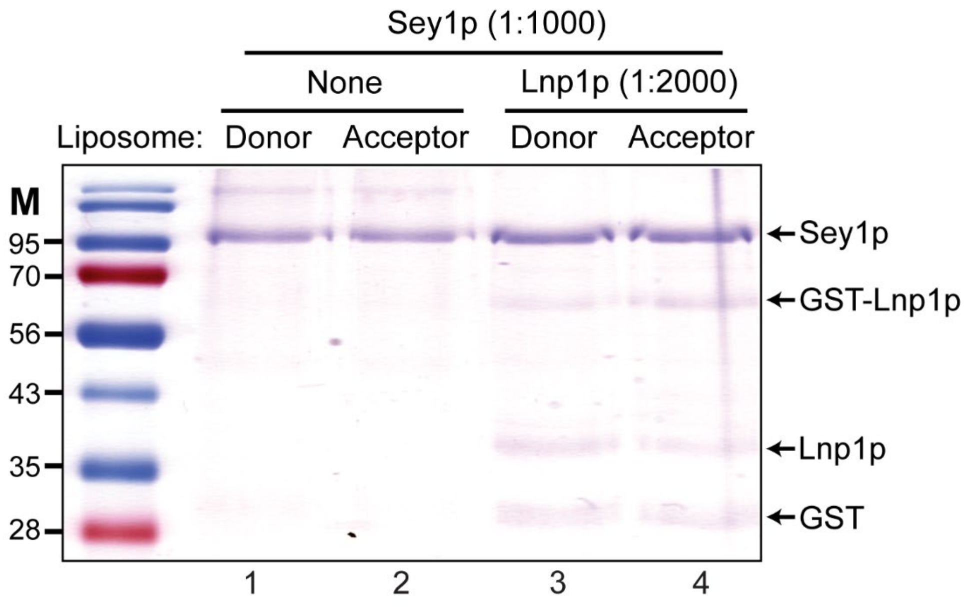

Figure S1. Addition of Lnp1p during liposome preparation does not affect the amount of Sey1p reconstituted into liposomes, related to Figure 2. Liposomes containing Sey1 alone or both Sey1p and Lnp1p were prepared as described in the ***STAR Methods***. Donor and acceptor proteoliposomes were solubilized in SDS sample buffer and analyzed by SDS-PAGE followed by Coomassie Brilliant Blue staining.

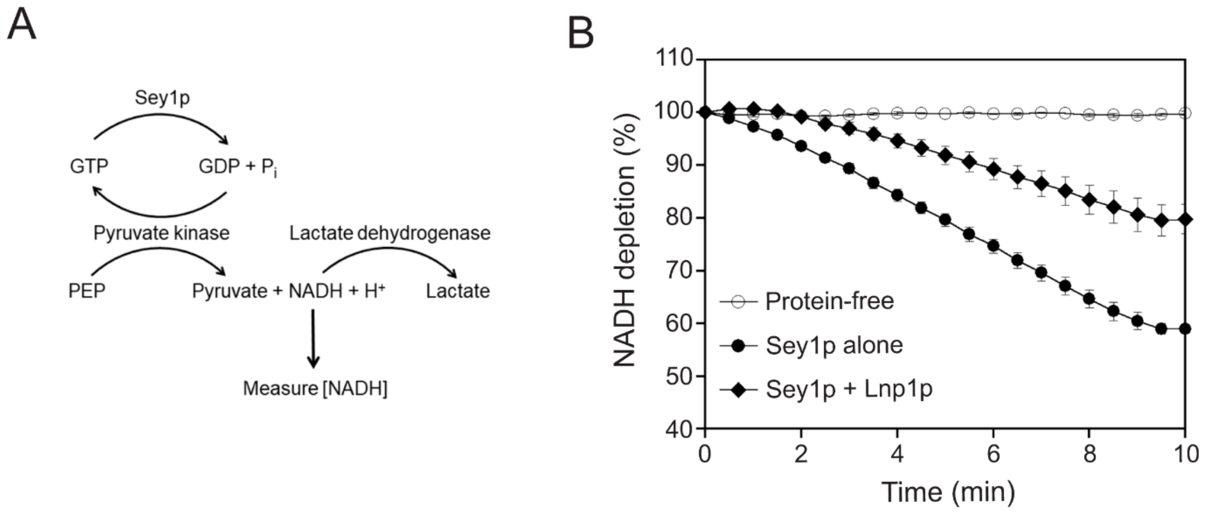

Figure S2. Lnp1p inhibits the GTPase activity of Sey1p, related to Figure 2. (A) Assay scheme. **(B)** The GTPase activity of Sey1p in the absence or presence of Lnp1p was determined using a continuous, coupled GTPase assay as depicted in (A). Proteoliposomes (1 mM) bearing Sey1p (1 μM) alone or both Sey1p (1 μM) and Lnp1p (0.5 μM) were incubated in reaction buffer (20 mM HEPES-NaOH, pH 7.4, and 150 mM NaCl) containing 60 μg/mL pyruvate kinase, 32 μg/mL lactate dehydrogenase, 4 mM phosphor(enol)pyruvate, 0.3 mM NADH, 2 mM GTP, and 10 mM $MgCl_2$. Depletion of NADH via its oxidation, which is directly proportional to GTP hydrolysis, was measured by monitoring the decrease in absorbance at 340 nm at 30°C every 30 sec for 10 min using a spectrometer through a 1 cm light path.

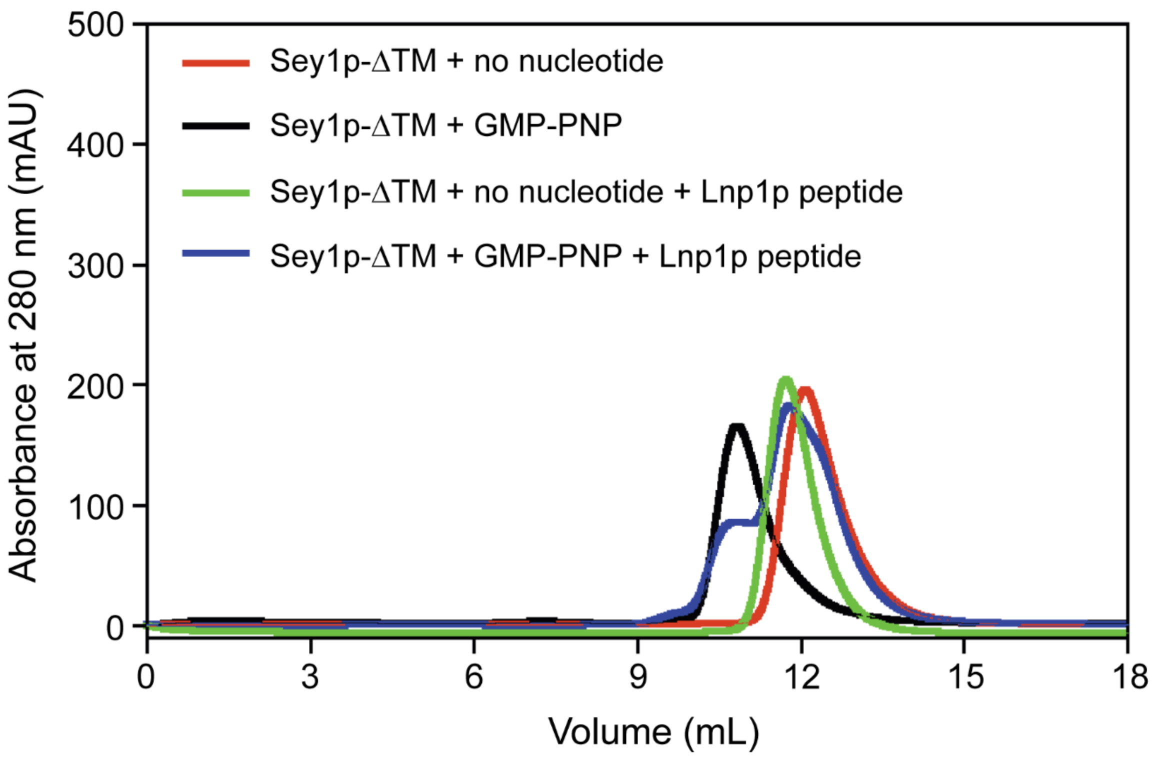

Figure S3. The Lnp1p peptide inhibits dimerization of Sey1p, related to Figure 6 and Figure 7. Size exclusion chromatography was performed with Sey1p-ΔTM in the absence or presence of the Lnp1p peptide. Sey1p-ΔTM was eluted from the column as monomers in the absence of nucleotide (red line), but migrated with a retention time consistent with homodimers in the presence of GMP-PNP (black line). In the presence of the Lnp1p peptide and GMP-PNP, the amount of Sey1p-ΔTM homodimers was markedly decreased (blue line).

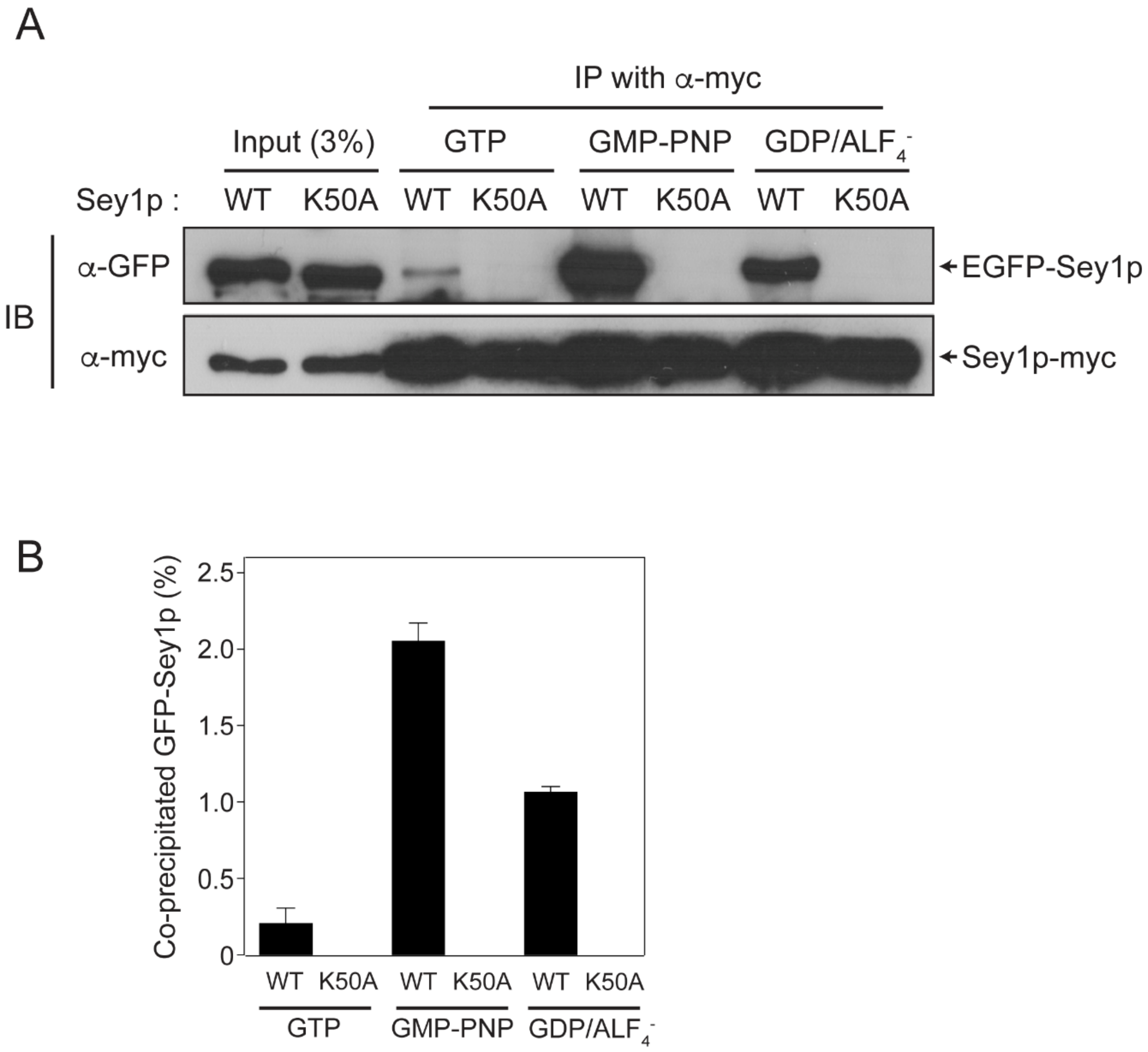

Figure S4. Formation of *trans*-Sey1p complexes requires nucleotides, related to Figure 4. The *trans*-Sey1p complex formation assay was performed in the absence or presence of GDP, GTP, or GTP analogs. Microsomes isolated from BJ-EGFP-Sey1p or BJ-EGFP-Sey1p-K50A were mixed with microsomes purified from BJ-Sey1p-myc or BJ-Sey1p-K50A-myc and incubated in the absence or presence of GTP or GTP analogs at 27°C. After 10 min, membranes were pelleted by centrifugation and solubilized on ice for 20 min. Detergent-insoluble material was removed by centrifugation at 4°C for 10 min, and Sey1p was precipitated using an anti-myc antibody and Protein A Sepharose. Co-precipitated EGFP-Sey1p was analyzed by SDS-PAGE followed by immunoblotting with an anti-GFP antibody. The immunoblots (A) are representative of three independent results, which are presented in a bar graph (B). Data represent the means ± SEM (error bars; n=3).

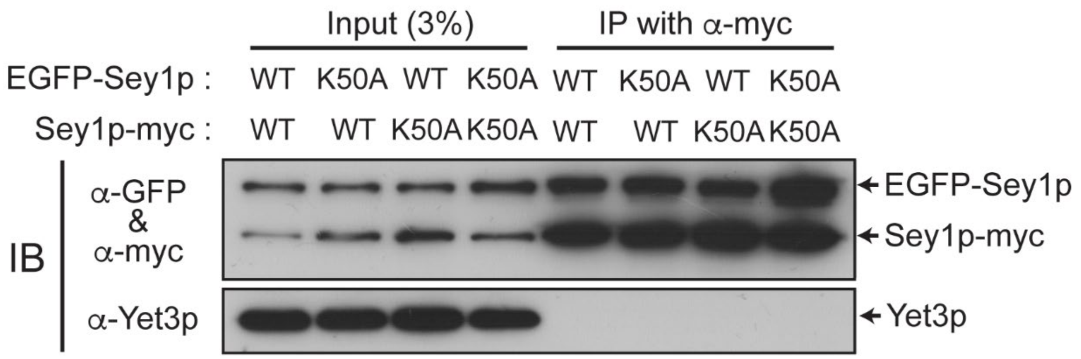

Figure S5. *Cis*-Sey1p complexes can form independently of GTP, related to Figure 5. Microsomes isolated from BJ-EGFP-Sey1p/Sey1p-myc, BJ-EGFP-Sey1p-K50A/Sey1p-myc, BJ-EGFP-Sey1p/Sey1p-K50A-myc, or BJ-EGFP-Sey1p-K50A/Sey1p-K50A-myc were solubilized, detergent-insoluble material was removed by centrifugation at 4°C for 10 min, and Sey1p-myc was precipitated using an anti-myc antibody and Protein A Sepharose. Co-precipitated EGFP-Sey1p was analyzed by SDS-PAGE followed by immunoblotting with an anti-GFP antibody.

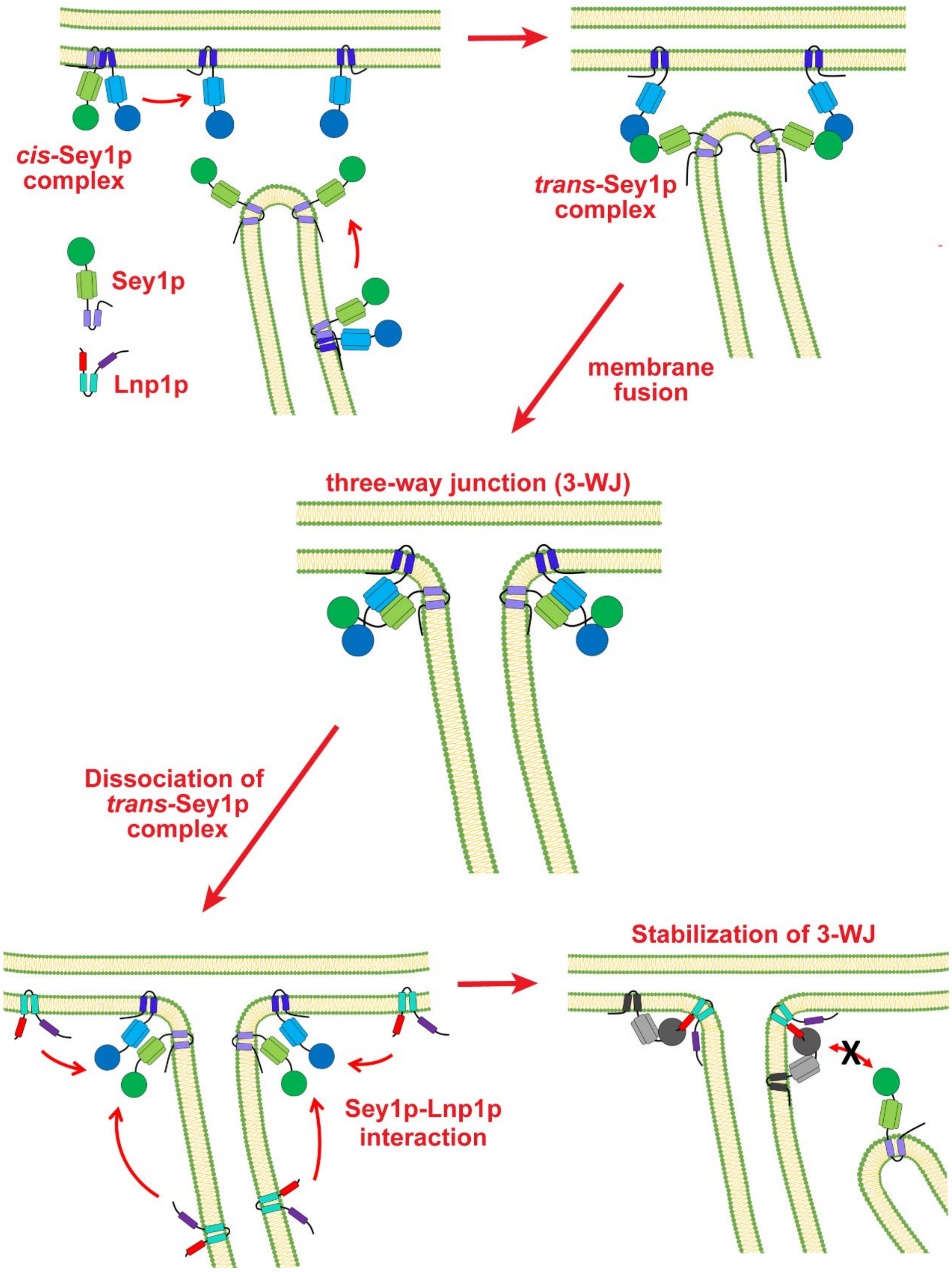

Supplementary Figure S6. A working model of how Lnp1p prevents excessive Sey1p-mediated fusion between ER tubules and thereby stabilizes preformed three-way junctions, related to Discussion. See text in *Discussion*.

Table S1. Yeast strains used in this study, related to STAR methods.

Strain	Genotype	Reference
BJ3505	MAT*a ura3-52 trp1-Δ101 lys2-208 gal2 can1 prb1-Δ1.6R pep4Δ::HIS3*	(Jones, 2002)[1]
BJ-Gluc1	BJ3505 *vam3*Δ::TRP1 bearing pYJ406-*ssZIP-GLuc1-HDEL*	(Lee et al., 2015)[2]
BJ-Gluc2	BJ3505 *vam3*Δ::TRP1 bearing pYJ406-*ssZIP-GLuc2-HDEL*	(Lee et al., 2015)[2]
BJ-Gluc1 *lnp1*Δ	BJ-Gluc1 with *lnp1*Δ::*URA3*	This study
BJ-Gluc2 *lnp1*Δ	BJ-Gluc2 with *lnp1*Δ::*URA3*	This study
BJ-Gluc1 *LNP1 O/E*	BJ-Gluc1 bearing pRS408-*pTDH3-LNP1*	This study
BJ-Gluc2 *LNP1 O/E*	BJ-Gluc2 bearing pRS408-*pTDH3-LNP1*	This study
BJ3505 *sey1*Δ	BJ3505 with *sey1*Δ::*KanMX4*	This study
BJ-Sey1p-myc	BJ3505 *sey1*Δ bearing pYJ406-*SEY1-myc*	This study
BJ-EGFP-Sey1p	BJ3505 *sey1*Δ bearing pYJ406-*EGFP-SEY1*	This study
BJ-Sey1p-K50A-myc	BJ3505 *sey1*Δ bearing pYJ406-*SEY1-K50A-myc*	This study
BJ-EGFP-Sey1p-K50A	BJ3505 *sey1*Δ bearing pYJ408-*EGFP-SEY1-K50A*	This study
BJ3505 *sey1*Δ *lnp1*Δ	BJ3505 *sey1*Δ *with lnp1*Δ::*URA3*	This study
BJ-Sey1p-myc *lnp1*Δ	BJ-Sey1p-myc with *lnp1*Δ::*URA3*	This study
BJ-EGFP-Sey1p *lnp1*Δ	BJ-EGFP-Sey1p with *lnp1*Δ::*URA3*	This study
BJ-Sey1p-myc *LNP1 O/E*	BJ-Sey1p-myc bearing pRS408-*pTDH3-LNP1*	This study
BJ-EGFP-Sey1p *LNP1 O/E*	BJ-EGFP-Sey1p bearing pRS408-*pTDH3-LNP1*	This study
BJ-Sey1p-myc/EGFP-Sey1p	BJ-Sey1p-myc bearing pYJ406-*EGFP-SEY1*	This study
BJ-Sey1p-myc/EGFP-Sey1p *lnp1*Δ	BJ-Sey1p-myc/EGFP-Sey1p with *lnp1*Δ::*URA3*	This study
BJ-Gluc1 *LNP1-3×HA*	BJ-Gluc1 *lnp1*Δ bearing pYJ408-*LNP1-3×HA*	This study

Table S1. (continued)

**Strain**	**Genotype**	**Reference**
BJ-Gluc2 *LNP1-3×HA*	BJ-Gluc2 *lnp1*Δ bearing pYJ408-*LNP1-3×HA*	This study
BJ-Gluc1 *LNP1-Δ186-278-3×HA*	BJ-Gluc1 *lnp1*Δ bearing pYJ408-*LNP1-Δ186-278-3×HA*	This study
BJ-Gluc2 *LNP1-Δ186-278-3×HA*	BJ-Gluc2 *lnp1*Δ bearing pYJ408-*LNP1-Δ186-278-3×HA*	This study
BJ-Gluc1 *LNP1-Δ2-52-3×HA*	BJ-Gluc1 *lnp1*Δ bearing pYJ408-*LNP1-Δ2-52-3×HA*	This study
BJ-Gluc2 *LNP1-Δ2-52-3×HA*	BJ-Gluc2 *lnp1*Δ bearing pYJ408-*LNP1-Δ2-52-3×HA*	This study
BJ-Sey1p-myc/*LNP1-3×HA*	BJ-Sey1p-myc *lnp1*Δ bearing pYJ408-*LNP1-3×HA*	This study
BJ-EGFP-Sey1p/*LNP1-3×HA*	BJ-EGFP-Sey1p *lnp1*Δ bearing pYJ408-*LNP1-3×HA*	This study
BJ-Sey1p-myc/*LNP1-Δ186-278-3×HA*	BJ-Sey1p-myc *lnp1*Δ bearing pYJ408-*LNP1-Δ186-278-3×HA*	This study
BJ-EGFP-Sey1p/*LNP1-Δ186-278-3×HA*	BJ-EGFP-Sey1p *lnp1*Δ bearing pYJ408-*LNP1-Δ186-278-3×HA*	This study
BJ-Sey1p-myc/*LNP1-Δ2-52-3×HA*	BJ-Sey1p-myc *lnp1*Δ bearing pYJ408- *LNP1-Δ2-52-3×HA*	This study
BJ-EGFP-Sey1p/*LNP1-Δ2-52-3×HA*	BJ-EGFP-Sey1p *lnp1*Δ bearing pYJ408-*LNP1-Δ2-52-3×HA*	This study

References

1. Jones, E.W. (2002). Vacuolar proteases and proteolytic artifacts in Saccharomyces cerevisiae. Methods Enzymol *351*, 127-150. 10.1016/s0076-6879(02)51844-9.

2. Lee, M., Ko, Y.J., Moon, Y., Han, M., Kim, H.W., Lee, S.H., Kang, K., and Jun, Y. (2015). SNAREs support atlastin-mediated homotypic ER fusion in Saccharomyces cerevisiae. Journal of Cell Biology *210*, 451-470. 10.1083/jcb.201501043.
